# B4GALNT4‐Mediated Glycosylation of PDK1 Activates the PI3K‐AKT Signaling Pathway to Promote Prostate Cancer Progression

**DOI:** 10.1002/advs.202511293

**Published:** 2026-02-16

**Authors:** Shaoqin Jiang, Mengqiang Li, Qingfu Su, Ruiling Dou, Shaoshan Lin, Jili Zhang, Xiaochen Sun, Haolan Yu, Wei Bao, Min Qu, Yan Wang, Chenghua Yang, Xu Gao

**Affiliations:** ^1^ Department of Urology Fujian Union Hospital Fujian Medical University Fuzhou China; ^2^ Department of Urology Changhai Hospital Second Military Medical University Shanghai China

**Keywords:** B4GALNT4, glycosylation, PDK1, PI3K‐AKT signaling pathway, prostate cancer

## Abstract

Abnormal glycosylation is a hallmark of cancer cells and plays a crucial role in tumor invasion and metastasis. However, the relationship between glycogenes and prostate cancer (PCa) remains poorly understood. This study aims to identify glycogenes involved in the onset and progression of PCa and to investigate the molecular mechanisms underlying their role. By integrating RNA‐seq data from multiple clinical cohorts (TCGA and CPGEA) and performing biochemical validation, we identified beta‐1,4‐N‐acetylgalactosaminyltransferase 4 (B4GALNT4) as a glycogene significantly associated with advanced pathological stages, higher Gleason scores, and poor prognosis in PCa patients. Furthermore, multivariate Cox regression analysis confirmed B4GALNT4 as an independent prognostic factor for survival. Mechanistically, we discovered that B4GALNT4 interacts with PDK1 and glycosylates it at residue N531. This N‐glycosylation stabilizes PDK1 by blocking its degradation, thereby activating the PI3K‐AKT signaling pathway. This signaling axis promotes PCa cell proliferation, migration, and invasion in vitro. Moreover, B4GALNT4 knockdown suppresses tumor growth in xenograft models and correlates with decreased PDK1 and p‐AKT levels in vivo. Our findings establish B4GALNT4 as a critical regulator of PCa progression through PDK1 glycosylation and PI3K‐AKT activation, suggesting that B4GALNT4 serves as both a prognostic biomarker and a potential therapeutic target for PCa.

## Graphical abstract

This study identifies B4GALNT4 as a key driver of prostate cancer (PCa). It shows that B4GALNT4 glycosylates PDK1 protein at the N531 site, which stabilizes the PDK1 and constitutively activates the PI3K‐AKT pathway. This mechanism promotes tumor cell proliferation, migration, and invasion. The findings establish the B4GALNT4‐PDK1 glycosylation axis as a novel therapeutic target and prognostic biomarker for PCa.

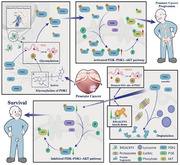

## 1 Introduction

Prostate cancer (PCa) is one of the most common malignancies in men, accounting for approximately 29% of new cancer cases in men in 2024 [1]. Although surgery and radiotherapy provide effective treatment options for patients with localized PCa, the treatment of advanced PCa still faces serious clinical challenges [2]. Androgen deprivation therapy (ADT) is the standard treatment option for advanced PCa, but due to the emergence of ADT resistance, 10% to 20% of patients with advanced PCa still progress to castration‐resistant prostate cancer (CRPC) within 5 years, resulting in a poor prognosis and a median overall survival (OS) of only about 3 years [3, 4]. Therefore, it is critical to explore new therapeutic targets and develop more effective treatment strategies for PCa.

Glycosylation is a widespread post‐translational modification regulated by glycosyltransferases (e.g., the B4GALNT family), nucleotide glycosynthesizing enzymes, transport proteins, and glycosidases. It plays a key regulatory role in a variety of physiological and pathological processes [5]. Studies show that aberrant glycosylation promotes tumor progression by affecting protein stability, and consequently leading to uncontrolled proliferation, EMT‐mediated invasion, and angiogenesis [6, 7, 8]. For example, B4GALT1 directly mediates N‑linked glycosylation of the PD‑L1 protein, thereby preventing the post‐translational degradation of PD‑L1 and maintaining its protein stability, which in turn promotes immune escape and tumor progression in lung adenocarcinoma cells [9]. Meanwhile, O‐GlcNAc modification of hepatocyte growth factor‐regulated tyrosine kinase substrate blocks its binding to PD‐L1 and inhibits the lysosomal degradation pathway of PD‐L1, leading to aberrant accumulation of PD‐L1 on the cell surface, thus also enhancing the malignant phenotype of the tumor [10]. In addition, glycosylation‐modified proteins (e.g., AFP, CA19‐9, and CA125) are routinely used as clinical tumor markers and play a key role in the early diagnosis of cancer [11, 12, 13]. Although glycosylation plays a crucial role in various cancers, systematic studies on glycosylation in PCa remain significantly underdeveloped.

Beta‐1,4‐N‐Acetyl‐Galactosaminyltransferase 4 (B4GALNT4) is a key glycosyltransferase that promotes the development of tumor‐associated proteins in a variety of cancers (e.g., breast, ovarian, and hepatocellular carcinomas) by regulating their glycosylation [14, 15, 16]. Our previous study identified elevated B4GALNT4 expression in PCa tissues [17], yet its functional role and molecular mechanisms in PCa remain poorly understood and warrant further investigation. Notably, B4GALNT4 catalyzes the transfer of N‐acetylgalactosamine (GalNAc) to N‐acetylglucosamine (GlcNAc), generating LacdiNAc (GalNAcβ1→4GlcNAc)—a potential diagnostic marker for PCa [18, 19]. This may indirectly indicate that B4GALNT4 has an important biological function in PCa, and further highlights the necessity of an in‐depth study of the role of B4GALNT4 in PCa.

The PI3K‐AKT signaling pathway plays a key role in the progression of PCa, and its aberrant activation is closely associated with tumor cell proliferation, metastasis, and treatment resistance [20]. Research has revealed that PTEN deficiency (observed in 40%–70% of metastatic PCa patients) can lead to sustained activation of the PI3K‐AKT pathway by lifting its negative regulation [21]. Additionally, high expression of the novel DNA repair enzyme NEIL3 can further activate the PI3K‐AKT axis, promoting PCa proliferation and metastasis [22]. Therefore, the PI3K‐AKT pathway, as a common hub for multiple oncogenic factors, plays a critical role in the development and progression of PCa.

As a key regulator of the PI3K‐AKT pathway, 3‐phosphatidylinositol‐dependent protein kinase‐1 (PDK1) can activate AKT through phosphorylation pathways, which in turn regulates the downstream pro‐survival signaling network [23]. In glioblastoma, PDK1 activates AKT by integrating upstream oncogenic signals (such as EGFR amplification/mutation and PTEN loss), driving invasive growth, inhibition of apoptosis, and angiogenesis in tumor cells [24, 25]. In breast cancer, PDK1‐mediated phosphorylation of AKT at the Thr308 site is a critical step driving abnormal proliferation and metastasis of cancer cells [26]. Therefore, PDK1 serves as a central component and a rate‐limiting step in PI3K signaling to AKT. In various cancer types, PDK1 is often overexpressed or exhibits enhanced activity, further amplifying oncogenic PI3K‐AKT signaling.

Building on this research background, this study aimed to systematically analyze the biological function of B4GALNT4 in PCa and its underlying molecular mechanism. Through integrative analysis of multiple transcriptomic databases, we identified *B4GALNT4* as a key glycosylation‐related gene that distinguishes high‐risk PCa patients. High B4GALNT4 expression was significantly associated with tumor stage, Gleason score, and poor prognosis in PCa. In‐depth mechanistic studies revealed that B4GALNT4 stabilizes PDK1 expression and continuously activates the PI3K‐AKT signaling pathway by mediating glycosylation at the N531 site of PDK1 protein. This study identifies a novel B4GALNT4‐PDK1 glycosylation axis that drives PCa progression through PDK1 stabilization, and it establishes a new paradigm for glycosylation‐mediated oncogenesis. Importantly, pharmacological inhibition of B4GALNT4‐mediated glycosylation may represent a promising therapeutic strategy for high‐risk PCa. Furthermore, targeting this axis may synergize with existing PI3K‐AKT inhibitors to overcome therapeutic resistance and improve outcomes in PCa.

## 2 Results

### 2.1 Glycogene B4GALNT4 is Identified to be Associated With High Risk Prostate Cancer

**FIGURE 1:**
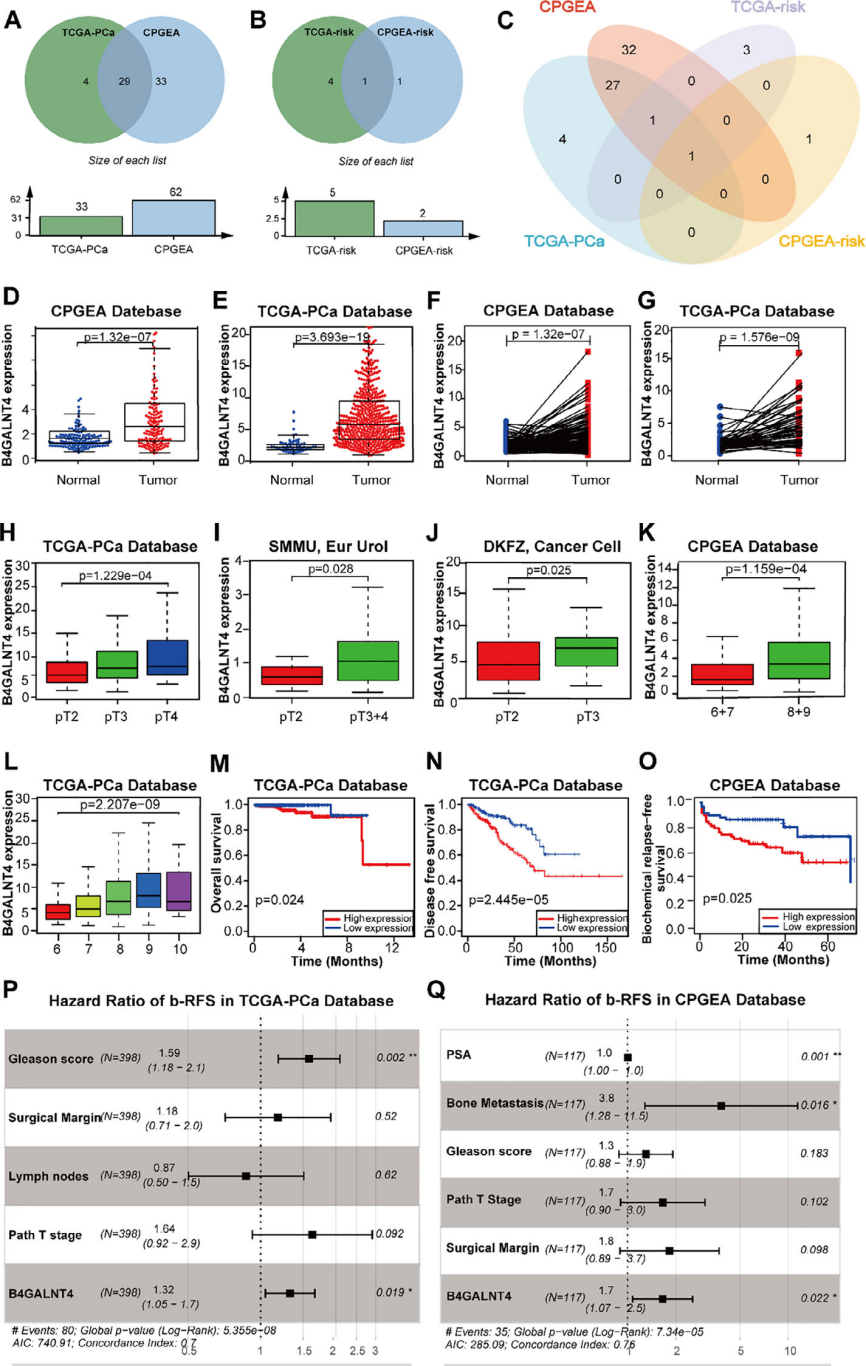
Glycogene *B4GALNT4* is identified to be associated with high‐risk prostate cancer. (A, B) Visualization of differentially expressed glycogenes between the tumor and normal groups in TCGA‐PCa and CPGEA databases (A); and between high‐risk and non‐high‐risk PCa groups (B). Intersected‐gene analysis disclosed subsets of differentially expressed glycogenes shared by the groups (A, n = 29; B, n = 1). (C) The Venn diagram illustrates the intersection of differentially expressed glycogenes between the tumor and normal groups, as well as the non‐high‐risk and high‐risk PCa groups in TCGA‐PCa and CPGEA databases. *B4GALNT4* was determined as the only glycogene significantly associated with PCa in this study. (D, E) Column plot of the mRNA expression of *B4GALNT4* in tumor and normal tissues from CPGEA (D) or TCGA‐PCa (E) database. (F‐G) Paired plot of the mRNA expression of *B4GALNT4* in PCa and adjacent tissues of patients from CPGEA (F) or TCGA‐PCa (G) database. (H‐J) Column plot of mRNA expression of *B4GALNT4* between different pathological stages of PCa patients in TCGA‐PCa (H), SMMU (I), DKFZ (J) database. (K, L) Column plot of mRNA expression of *B4GALNT4* between different Gleason scores of PCa patients in CPGEA (K) and TCGA‐PCa (L) databases. (M, N) High *B4GALNT4* expression in TCGA‐PCa was positively associated with worse OS (M) and DFS (N). (O) High *B4GALNT4* expression in the CPGEA database was positively associated with worse b‐RFS. (P, Q) Forest plot showing the b‐RFS prognostic score for each clinical parameter in a multivariate Cox regression analysis in TCGA‐PCa (P) or CPGEA (Q) database. Data are presented as HR (95% CI) (^*^, *p* < 0.05; ^**^, *p* < 0.01). OS, overall survival; DFS, disease‐free survival; b‐RFS, biochemical relapse‐free survival; Path T stage: Pathological T stage.

In this study, we extracted 185 human glycogenes from the Glycogene Database (GGDB) (Table S1). In our in‐house Chinese Prostate Cancer Genome and Epigenome Atlas (CPGEA, n = 272), we identified 62 significantly differentially expressed glycogenes (DEGGs) between tumor and normal tissues of PCa (Figure S1A). In the Cancer Genome Atlas‐PCa (TCGA‐PCa, n = 551), we identified 33 DEGGs (Figure S1B), 29 of which were shared between the two databases (Figure 1A, Table S2).

We then stratified PCa patients in the CPGEA (n = 123) and TCGA‐PCa (n = 404) cohorts into high‐risk and non‐high‐risk groups according to the 2023 European Association of Urology (EAU) PCa risk classification criteria (Table S3) [27]. Survival analysis revealed that high‐risk patients in the CPGEA‐risk database had a shorter biochemical relapse‐free survival (b‐RFS) (Figure S1C), while in the TCGA‐risk database, high‐risk patients had worse overall survival (OS) (Figure S1D). Differential analysis of 185 glycogenes was performed between the high‐risk and non‐high‐risk groups and visualized using heatmaps in the CPGEA‐risk and TCGA‐risk databases (Figure S1E,F). Among these genes, two were identified as DEGGs in the CPGEA‐risk database and five in the TCGA‐risk database, with one glycogene common to both (Figure 1B, Table S4).

After taking the intersection of the aforementioned four gene sets, *B4GALNT4* was the only common DEGG (Figure 1C). Consequently, *B4GALNT4* was selected as a glycogene for further studies. The glycogene screening process is detailed in Figure S2.

*B4GALNT4* mRNA expression was significantly higher in PCa tissues from the CPGEA and TCGA‐PCa database compared to normal prostate tissues (Figure 1D,E). This finding was further confirmed by paired analysis of PCa and adjacent non‐cancerous prostate tissues (Figure 1F,G). Analysis of various PCa databases revealed that increased *B4GALNT4* expression was significantly associated with more advanced pathological stages (Figure 1H–J) and higher Gleason scores (GS) (Figure 1K,L). These results suggest that *B4GALNT4* may serve as a key marker of poor prognosis in PCa. Furthermore, survival analysis corroborated this conclusion. In the TCGA‐PCa database, high *B4GALNT4* expression was significantly correlated with poorer OS and disease‐free survival (DFS) (Figure 1M,N). In the CPGEA database, high *B4GALNT4* expression was linked to poorer b‐RFS (Figure 1O). Additionally, multivariate Cox regression analysis revealed that *B4GALNT4* mRNA expression was an independent risk factor for b‐RFS in PCa patients (Figure 1P,Q, Tables S5 and S6). These findings indicate that *B4GALNT4* expression is strongly correlated with prostate cancer progression.

### 2.2 B4GALNT4 Promotes the Malignant Characteristics of PCa Cells

**FIGURE 2:**
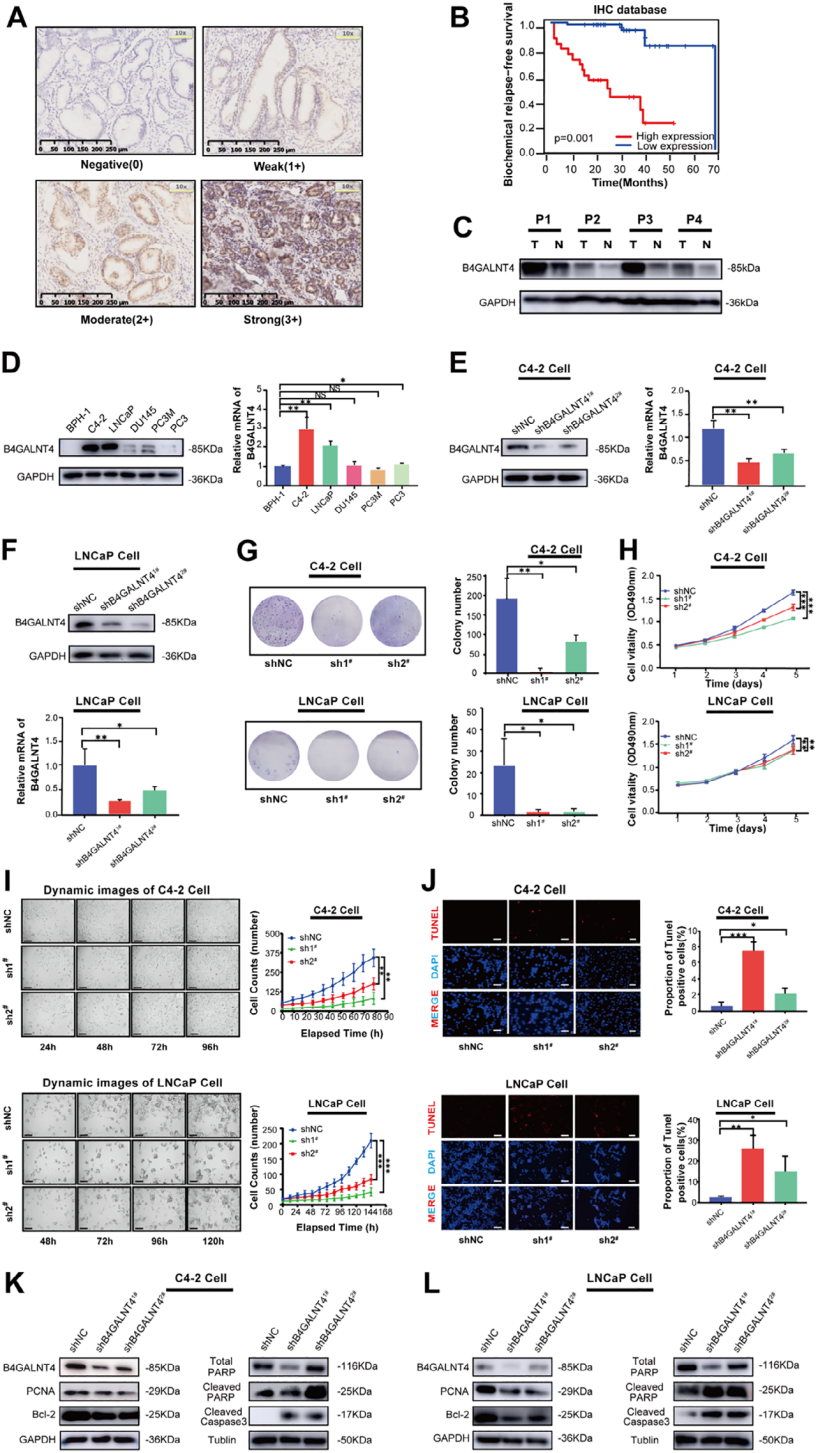
B4GALNT4 promotes the malignant characteristics of PCa cells. (A) The representative IHC staining images demonstrated the expression of B4GALNT4 in PCa tumor tissues (Scale bar: 250 µm). (B) The relationship between B4GALNT4 expression and b‐RFS was assessed using the median IHC score of B4GALNT4 in tumor tissues as the cutoff point. High expression: n = 25, low expression: n = 42. (C), Western blotting of B4GALNT4 in tumor (T) and adjacent normal (N) tissues of fresh prostate tumors from Changhai Hospital. (D) Protein and mRNA expression of B4GALNT4 was assessed in five human PCa cell lines (C4‐2, LNCaP, DU145, PC3M, and PC3) and a human benign prostate cell line (BPH‐1) using western blotting (left) and qRT‐PCR (right). (E, F) Western blotting and qRT‐PCR analysis of B4GALNT4 protein and mRNA in control (shNC) or *B4GALNT4‐*knockdown (sh1^#^, sh2^#^) C4‐2 (E) cells or LNCaP (F) cells. (G) Clone formation assay for C4‐2 and LNCaP cells with (sh1^#^, sh2^#^) or without (shNC) *B4GALNT4* knockdown. (H) CCK‐8 assay to evaluate the effect of *B4GALNT4* knockdown (sh1^#^, sh2^#^) on the proliferative ability of C4‐2 or LNCaP cells. (I) Proliferation of C4‐2 or LNCaP cells following *B4GALNT4* knockdown (sh1^#^, sh2^#^) was monitored using a live‐cell dynamic imaging assay. Scale bar: 50 µm. Representative images at the indicated time points are shown. (J) TUNEL assay to assess the effect of *B4GALNT4* knockdown (sh1^#^, sh2^#^) on apoptosis in C4‐2 and LNCaP cells. Scale bar: 50 µm. (K, L) Western blotting for the protein expression of PCNA, Bcl‐2, total PARP, cleaved PARP, and cleaved Caspase 3 in *B4GALNT4* knockdown (sh1^#^, sh2^#^) of C4‐2 (K) and LNCaP (L) cells. sh1^#^, shB4GALNT4^1#^; sh2^#^, shB4GALNT4^2#^. NS, *p* > 0.05, ^*^, *p* < 0.05; ^**^, *p* < 0.01; ^***^, *p* < 0.001.

We further investigated the expression and function of B4GALNT4 in PCa through experimental validation. Specifically, Immunohistochemistry (IHC) staining of 67 clinical PCa tissue samples revealed that B4GALNT4 protein was predominantly expressed in the cytoplasm of prostatic acinar epithelial cells (Figure 2A). Based on the IHC results, PCa patients were classified into two groups with 25 cases in the high B4GALNT4 expression group and 42 in the low expression group. The b‐RFS was significantly lower in the high B4GALNT4 expression group (Figure 2B). Moreover, patients in the high B4GALNT4 expression group exhibited higher PSA levels, GS, extraprostatic invasion, seminal vesicle invasion, and pathological T stage (Table S7). Western blotting analysis revealed that B4GALNT4 protein expression was significantly higher in PCa tissues than in adjacent normal tissues (Figure 2C). Collectively, these findings suggest that high B4GALNT4 expression is linked to PCa progression and poor prognosis, making it a potential biomarker. Additionally, we also assessed B4GALNT4 expression in five PCa cell lines (C4‐2, LNCaP, DU145, PC3M, PC3) and one benign prostate cell line (BPH‐1). In both C4‐2 and LNCaP cell lines, B4GALNT4 was highly expressed at the mRNA and protein levels (Figure 2D). Subsequently, we knocked down B4GALNT4 expression using two shRNAs named shB4GALNT4^1#^ (sh1^#^) and shB4GALNT4^2#^ (sh2^#^) in both cell lines (Figure 2E,F). B4GALNT4 knockdown significantly inhibited colony formation (Figure 2G) and proliferation (Figure 2H,I) of C4‐2 and LNCaP cells compared to the control (shNC). TUNEL assay showed that downregulation of B4GALNT4 significantly induced apoptosis in C4‐2 and LNCaP cells (Figure 2J). Following B4GALNT4 downregulation, the expression levels of the proliferation marker PCNA and the anti‐apoptotic protein Bcl‐2 were significantly decreased, while the expression levels of apoptosis markers cleaved PARP and cleaved Caspase3 were significantly increased in C4‐2 and LNCaP cells (Figure 2K,L). Moreover, B4GALNT4 downregulation significantly inhibited the invasion and migration abilities of C4‐2 and LNCaP cells (Figure S3A–D).

### 2.3 B4GALNT4 Interacts With PDK1 and Activates the PI3K‐AKT Signaling Pathway in PCa Cells

**FIGURE 3:**
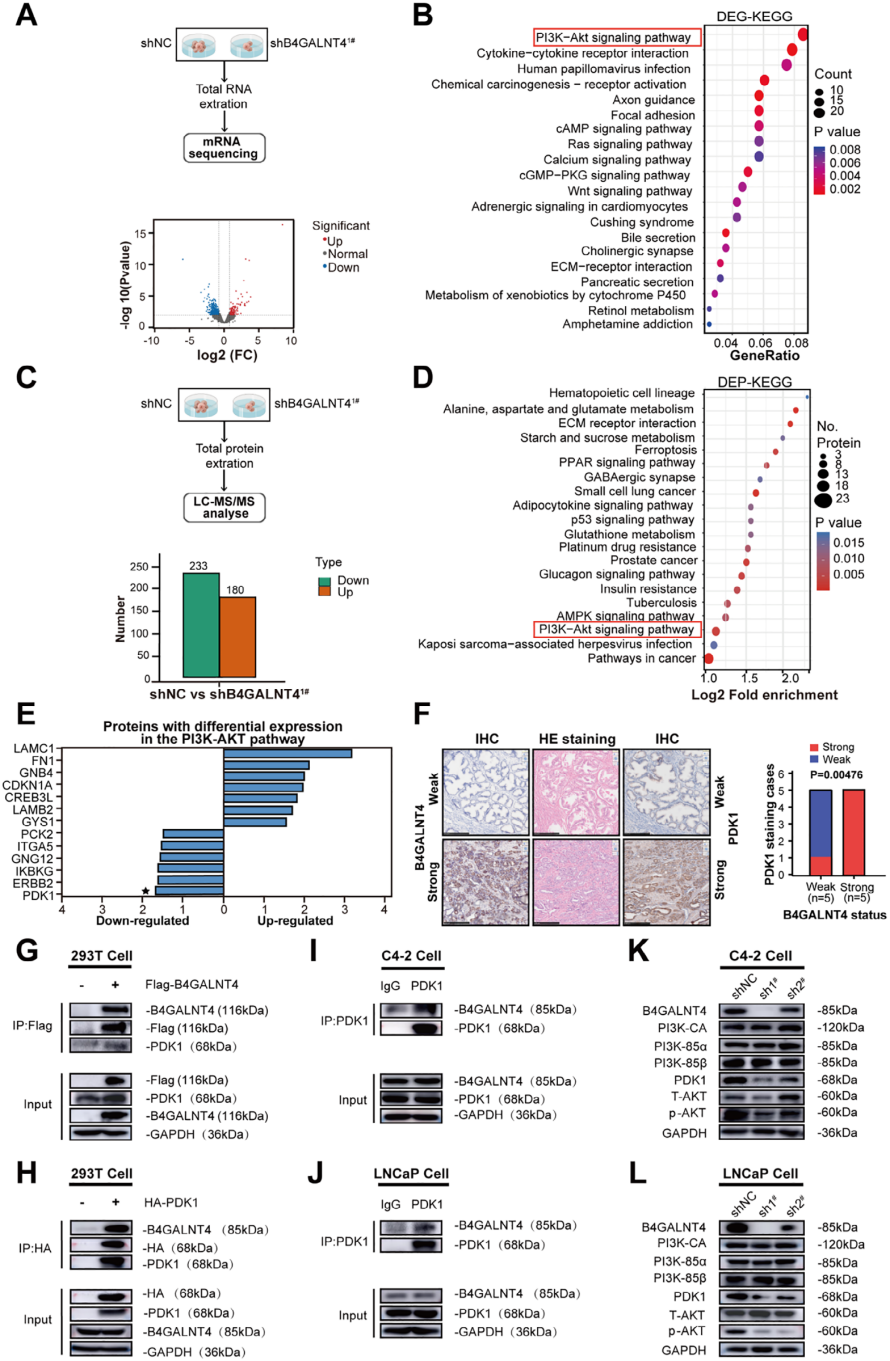
B4GALNT4 interacts with PDK1 and activates the PI3K‐AKT signaling pathway in PCa cells. (A) Workflow for RNA transcriptome sequencing of C4‐2‐shB4GALNT4^1#^ and C4‐2‐shNC cells. Volcano plot displays differentially expressed genes (DEGs). Red dots indicate upregulated genes and blue dots indicate downregulated genes. Screening criteria for significance were log2FC≥2 and FDR<0.01. (B) KEGG pathway analysis of enriched pathways of DEGs between C4‐2‐shB4GALNT4^1#^ and C4‐2‐shNC cells. (C) Workflow for LC‐MS/MS protein profiling of C4‐2‐shB4GALNT4^1#^ and C4‐2‐shNC cells. The column plot shows differentially expressed proteins (DEPs). The screening criteria were as follows: FC>1.5 for up‐regulation, and FC<1/1.5 for down‐regulation. (D) KEGG pathway analysis of enriched pathways of DEPs between C4‐2‐shB4GALNT4^1#^ and C4‐2‐shNC cells. (E) DEPs related to the PI3K‐AKT signaling pathway identified by quantitative proteomics analysis, comparing C4‐2‐shB4GALNT4^1#^ cells with C4‐2‐shNC cells. (F) Representative HE and IHC staining images of clinical PCa tissues illustrate the difference in PDK1 protein expression between tumors with strong (n = 5) and weak (n = 5) B4GALNT4 expression (Scale bar: 250 µm). (G, H) Western blotting analysis of co‐immunoprecipitation assays shows the interaction between B4GALNT4 and PDK1 in 293T cells overexpressing Flag‐B4GALNT4 (G) and HA‐PDK1 (H), respectively. Overexpression of Flag‐B4GALNT4 in 293T cells resulted in the detection of a full‐length B4GALNT4 band at approximately 116 kDa (G). Endogenous B4GALNT4 was predominantly detected at approximately 85 kDa (H). (I, J) Western blotting analysis of co‐immunoprecipitation assays showing the interaction between B4GALNT4 and PDK1 in C4‐2 cells (I) or LNCaP cells (J). (K, L) Western blotting of PI3K‐AKT signaling proteins in C4‐2(K) and LNCaP cells (L) transfected with B4GALNT4 shRNAs (sh1^#^, sh2^#^) or control shRNA (shNC). sh1^#^, shB4GALNT4^1#^; sh2^#^, shB4GALNT4^2#^. log2FC: log2 fold change; FDR: false discovery rate.

We investigated how B4GALNT4 promotes the malignant phenotype of PCa cells. To do this, we performed mRNA sequencing (Figure 3A,B) and proteomic analysis (Figure 3C,D) on C4‐2‐shNC and C4‐2‐shB4GALNT4^1#^ cells. Detailed RNA‐sequencing and proteomic data are provided in the Tables S8 and S9. Differential analysis identified 638 differentially expressed genes (DEGs, Figure 3A) and 413 differentially expressed proteins (DEPs, Figure 3C). KEGG pathway analysis revealed significant enrichment of DEGs and DEPs in the PI3K‐AKT signaling pathway (Figure 3B,D). Further proteomic analysis revealed that knockdown of B4GALNT4 most significantly downregulated PDK1 protein, a key protein in the PI3K‐AKT pathway (Figure 3E). However, corresponding RNA‐seq data showed no statistically significant change in PDK1 mRNA levels (Figure S4A), suggesting that B4GALNT4 may regulate PDK1 at the protein level via a post‐translational mechanism. IHC results confirmed cytoplasmic co‐localization of PDK1 and B4GALNT4 in PCa cells, with their protein levels positively correlated (Figure 3F).

We then explored the interaction between B4GALNT4 and PDK1 in PCa cells. Co‐immunoprecipitation assay showed that both exogenous and endogenous B4GALNT4 protein interacted with PDK1 protein (Figure 3G–J). Knockdown of B4GALNT4 in C4‐2 or LNCaP cells (shB4GALNT4^1#^, shB4GALNT4^2#^) significantly down‐regulated PDK1 expression and its downstream PI3K‐AKT pathway, while PI3K‐related upstream proteinBs (PI3K‐CA, PI3K‐85α, PI3K‐85β) showed no significant changes (Figure 3K,L).

### 2.4 PDK1 Overexpression Restores the Prostate Cancer Suppressed by B4GALNT4 Knockdown

**FIGURE 4:**
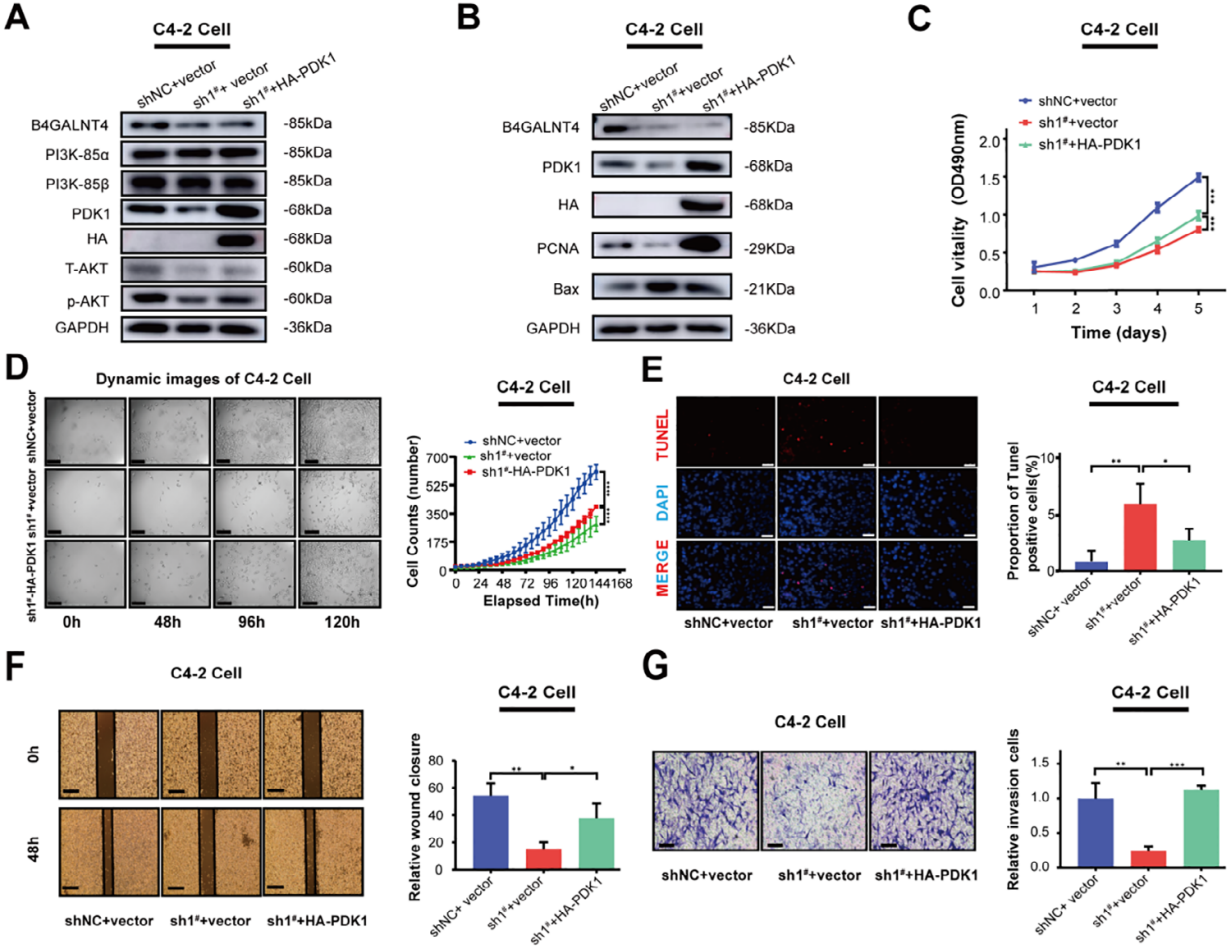
PDK1 overexpression restores the prostate cancer suppressed by B4GALNT4 knockdown. (A, B) Western blotting analysis of the indicated proteins was performed in B4GALNT4 knockdown C4‐2 cells overexpressing HA‐PDK1 and in control C4‐2 cells. (C) CCK‐8 assay analyzed proliferation alterations of different cell groups: C4‐2‐shNC+vector, C4‐2‐sh1^#^+vector, sh1^#^+HA‐PDK1. (D) Cell proliferation assay of different cell groups by Live‐cell imaging: C4‐2‐shNC+vector, C4‐2‐sh1^#^+vector, sh1^#^+HA‐PDK1. Representative images captured at 0 h, 48 h, 96 h, 120 h (Scale bar: 50 µm). (E) TUNEL assay of different cell groups: C4‐2‐shNC+vector, C4‐2‐sh1^#^+vector, sh1^#^+HA‐PDK1 (Scale bar: 50 µm). (F, G) Migration (F) and invasion (G) assays performed in different cell groups: C4‐2‐shNC+vector, C4‐2‐sh1^#^+vector, sh1^#^+HA‐PDK1. Representative images and statistical analyses were shown (Scale bar: 50 µm). Experiments were conducted in triplicate, and data are presented as mean ± SD (^*^, *p* < 0.05; ^**^, *p* < 0.01; ^***^, *p* < 0.001; NS, *p* > 0.05). shNC, the negative control shRNA; sh1^#^, shB4GALNT4^1#^.

To explore PDK1's role in PCa progression, we overexpressed PDK1 in C4‐2‐shB4GALNT4^1#^ cells and found that PDK1 overexpression partially reversed the phenotypic changes caused by B4GALNT4 knockdown, as evidenced by increased p‐AKT and PCNA expression and decreased Bax (apoptosis inducer) expression (Figure 4A,B). PDK1 overexpression also reversed the inhibitory effects of B4GALNT4 knockdown on tumor cell proliferation (Figure 4C,D), apoptosis (Figure 4E), migration (Figure 4F), and invasion (Figure 4G). The specific PDK1 inhibitor BX‐795 inhibited the proliferation of C4‐2 and LNCaP cells with IC50 values of 9.864 and 2.942 µm, respectively. However, after B4GALNT4 knockdown, the proliferation curve shifted to the right with IC50 values of 25.35 and 6.019 µm, indicating reduced sensitivity to BX‐795 (Figure S4B,C). Our results suggest that B4GALNT4 drives PCa progression by regulating the PI3K‐AKT pathway through PDK1.

### 2.5 B4GALNT4 Stabilizes PDK1 Through Post‐Translational Glycosylation

**FIGURE 5:**
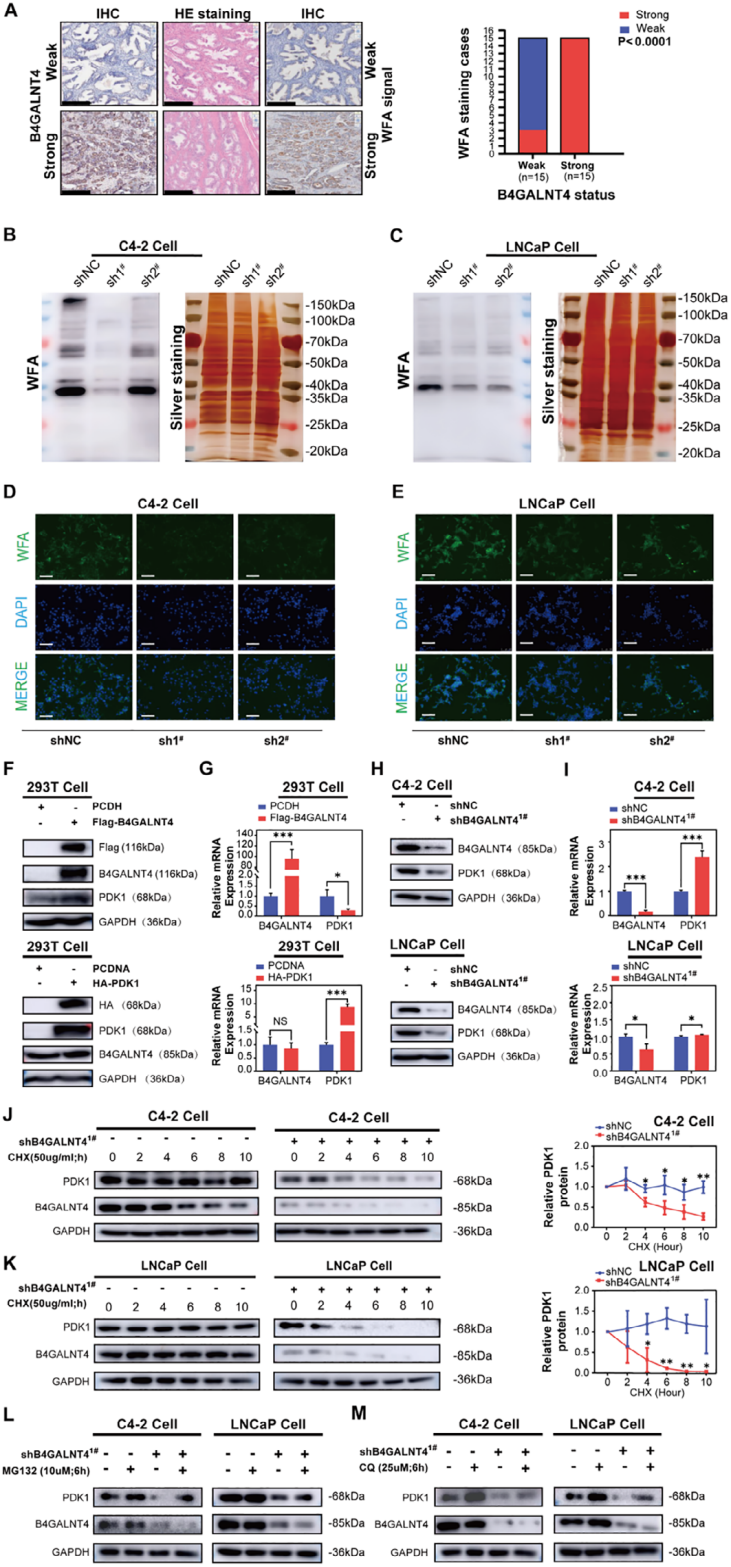
B4GALNT4 stabilizes PDK1 through post‐translational glycosylation. (A) IHC staining images of B4GALNT4 and WFA staining for GalNAc‐containing glycans in clinical PCa tissues, classified into strong and weak expression groups based on B4GALNT4 protein intensity (scale bar: 250 µm). The right‐hand bar graph illustrates that higher B4GALNT4 protein levels are associated with increased WFA staining intensity. (B, C) Lectin blot analysis with biotinylated WFA of lysates from C4‐2 (B) or LNCaP (C) cells transfected with control (shNC) or B4GALNT4‐targeting shRNAs (sh1^#^, sh2^#^). Silver staining of the same gel is shown as a loading control. (D, E) WFA immunofluorescence staining (green) in C4‐2 (D) and LNCaP (E) cells after B4GALNT4 knockdown, confirming the reduction in cell‐associated GalNAc‐containing glycans. Nuclei are counterstained with DAPI (blue) (scale bar: 50 µm). (F, G) Western blotting (F) and qRT‐PCR (G) of B4GALNT4 and PDK1 expression in 293T cells transfected with Flag‐B4GALNT4 or HA‐PDK1, respectively. Overexpression of Flag‐B4GALNT4 in 293T cells resulted in the detection of a full‐length B4GALNT4 band at approximately 116 kDa (F). Endogenous B4GALNT4 was predominantly detected at approximately 85 kDa. (H‐I) Western blotting (H) and qRT‐PCR (I) of B4GALNT4 and PDK1 expression in B4GALNT4‐knockdown C4‐2 or LNCaP cells. (J, K) Western blotting of PDK1 and B4GALNT4 in C4‐2 (J) and LNCaP (K) cells treated with CHX. Quantification of PDK1 expression is shown on the right. (L, M) Western blotting of PDK1 and B4GALNT4 in B4GALNT4‐knockdown C4‐2 and LNCaP cells treated with MG132 (L) or CQ (M). Experiments were conducted in triplicate, and data are presented as mean ± SD (^*^, *p* < 0.05; ^**^, *p* < 0.01; ^***^, *p* < 0.001; NS, *p* > 0.05). sh1^#^, shB4GALNT4^1#^; sh2^#^, shB4GALNT4^2#^.

Wisteria floribunda agglutinin (WFA) is a lectin that specifically recognizes glycans ending with N‐acetylgalactosamine (GalNAc) [28, 29], a key saccharide in both O‐ and N‐linked glycoproteins. To assess the global glycosylation changes mediated by B4GALNT4, we first performed IHC analysis on clinical PCa tissues. This analysis revealed a positive correlation between B4GALNT4 protein levels and WFA staining intensity (Figure 5A), suggesting that B4GALNT4 expression is associated with WFA‐reactive GalNAc‐containing glycans. Additionally, to investigate how B4GALNT4 modulates the expression of GalNAc‐terminated glycans in PCa cells, we performed lectin blotting analyses. Knocking down B4GALNT4 (sh1^#^ and sh2^#^) in C4‐2 and LNCaP cells significantly reduced the intensity of multiple WFA‐reactive bands compared to the control (shNC) (Figure 5B,C). Consistently, WFA‐based immunofluorescence staining also showed a substantial decrease in fluorescent signal upon B4GALNT4 knockdown in both cell lines (Figure 5D,E). This concordant reduction in WFA reactivity, demonstrated by two independent assays, indicated that B4GALNT4 knockdown led to a global decrease in GalNAc‐containing glycans, confirming its essential role in sustaining global GalNAcylation levels in these PCa cells.

Next, we investigated whether PDK1 is a substrate for B4GALNT4 glycosylation. Initially, we used 293T cells for preliminary validation. Ectopic overexpression of B4GALNT4 (Flag‐B4GALNT4) in 293T cells resulted in an increase in PDK1 protein expression; however, there was no increase in the mRNA expression of PDK1. In contrast, when PDK1 was overexpressed in 293T cells, no significant alterations were observed in the expression of B4GALNT4 at either the mRNA or protein levels (Figure 5F,G). We next moved to C4‐2 and LNCaP cells to verify the findings in a disease‐relevant context. Notably, knockdown of B4GALNT4 (shB4GALNT4^1#^) in PCa cells downregulated PDK1 protein expression without reducing its mRNA levels (Figure 5H,I). These results indicate that B4GALNT4 may modulate PDK1 protein via post‐translational glycosylation. This phenomenon was not only observed in 293T cells but is also reproducible in PCa cells (C4‐2 and LNCaP).

We performed a cycloheximide (CHX) chase assay to directly test this hypothesis and quantify its effect on protein half‐life. We found that knockdown of B4GALNT4 significantly accelerated the degradation of PDK1 protein, markedly shortening its half‐life in both C4‐2 and LNCaP cells (Figure 5J,K), which confirmed that B4GALNT4 enhanced PDK1 protein stability.

To elucidate the specific degradation pathway of PDK1, we treated cells with the proteasome inhibitor MG132 at 10 µm for 6 h or the lysosome inhibitor chloroquine (CQ) at 25 µm for 6 h. As shown in Figure 5L,M, knockdown of B4GALNT4 in C4‐2 or LNCaP cells led to reduced PDK1 expression. Treatment with MG132 or CQ restored PDK1 levels, indicating that B4GALNT4 catalyzed post‐translational glycosylation protects PDK1 from proteasomal and lysosomal degradation.

### 2.6 B4GALNT4 Mediates N‐Glycosylation of PDK1 at the N531 Site

**FIGURE 6:**
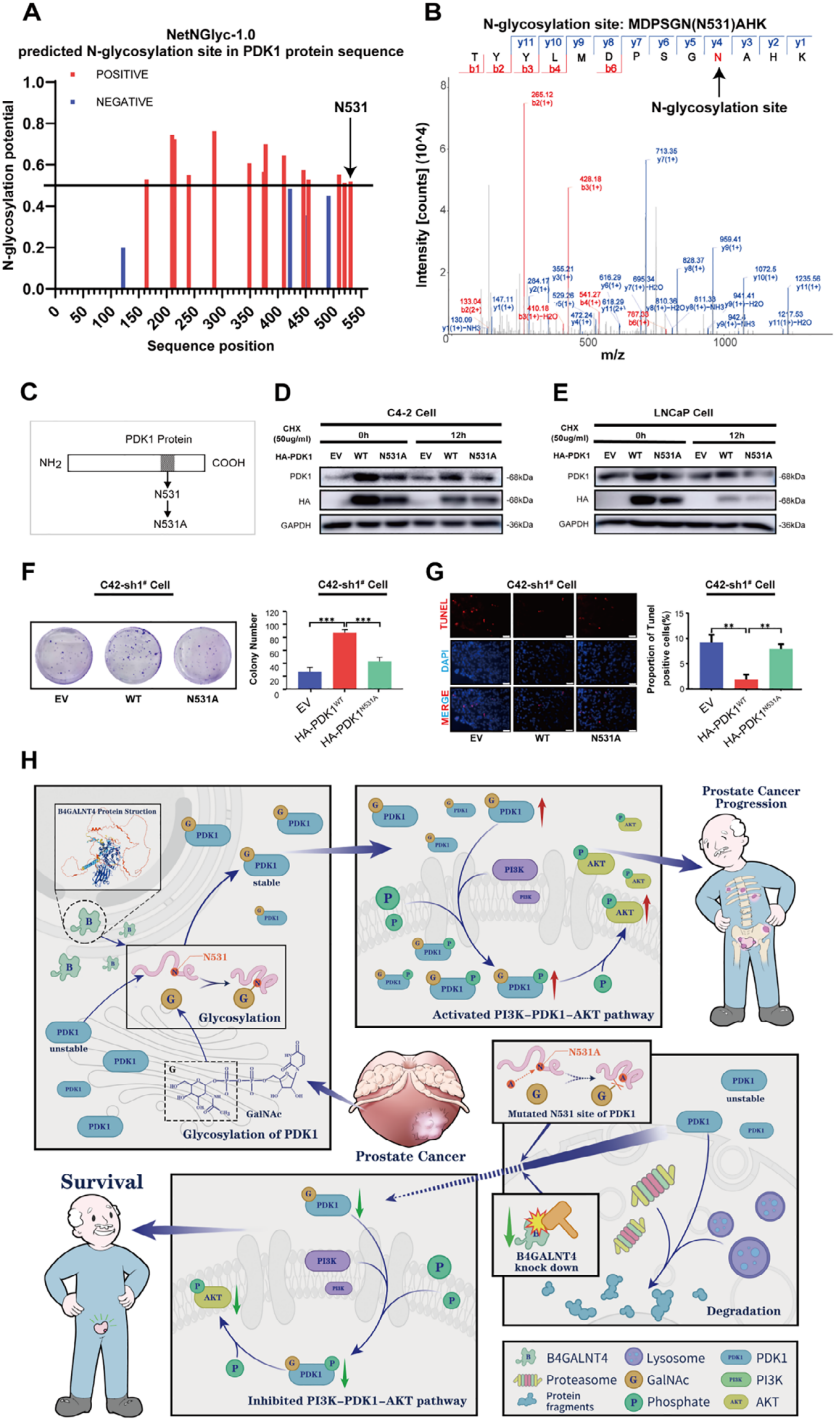
B4GALNT4 mediates N‐glycosylation of PDK1 at the N531 site. (A) N‐glycosylation site prediction of PDK1 using the NetNGlyc‐1.0 tool from the online protein database prediction website (https://acgg.asia/ggdb2). Red lines indicate positive sites with a high probability of N‐glycosylation. Blue lines indicate negative sites for low probability of N‐glycosylation. (B) LC‐MS/MS analysis detected the N531 site as the major site for N‐glycosylation of PDK1 protein. (C) Model view of the N531A mutant, in which the asparagine(N) at position 531 of PDK1 was mutated to alanine (A). (D, E) Western blotting of PDK1 in C4‐2 cells (D) or LNCaP cells (E) that were overexpressed with empty vector(EV), PDK1 wild‐type (HA‐PDK1^WT^), or N531 mutant of PDK1 protein (HA‐PDK1^N531A^), and treated with the protein synthesis inhibitor CHX (50 µg/mL). (F, G) Clone formation assay (F), and TUNEL staining assay (G) of B4GALNT4 downregulated C4‐2 cells overexpressing EV, HA‐PDK1^WT^, or HA‐PDK1^N531A^. The quantification of each assay is shown right. Scale bar: 50 µm. Experiments were conducted in triplicate, and data are presented as mean ± SD (^*^, *p* < 0.05; ^**^, *p* < 0.01; ^***^, *p* < 0.001; NS, *p* > 0.05). (H) Summary illustration. Glycosyltransferase B4GALNT4 mediates the glycosylation of PDK1 at the N531 site, increasing the stability of PDK1 and thereby activating the PI3K‐AKT signaling pathway in PCa cells, promoting the malignant characteristics of prostate cancer cells. However, knocking down B4GALNT4 or mutating the N531 site of the PDK1 protein decreases the stability of PDK1, inhibits the PI3K‐AKT signaling pathway in PCa cells, and suppresses the malignant characteristics of tumor cells, thereby improving patient survival. sh1^#^, shB4GALNT4^1#^; sh2^#^, shB4GALNT4^2#^.

To identify the specific glycosylation sites on the PDK1 protein, we first performed in silico prediction using the NetNGlyc‐1.0 and NetOGlyc‐4.0 tools. A prediction score greater than 0.5 was considered indicative of a positive glycosylation site, while a score below this threshold was deemed negative. The analysis revealed that NetNGlyc‐1.0 predicted 14 potential N‐glycosylation sites on PDK1 (Figure 6A), and NetOGlyc‐4.0 predicted 32 O‐glycosylation sites (Figure S4D), indicating a high theoretical propensity for glycosylation of PDK1. The complete prediction results are provided in Table S10.

To experimentally map glycosylation sites, endogenous PDK1 was immunoprecipitated from C4‐2 cells. Based on LC‐MS/MS analysis of the PDK1 band, 20 unique peptides were identified (33% sequence coverage; Table S11). A targeted post‐translational modification (PTM)‐enriched search, incorporating deamidation (+2.99 Da, Asparagine) as a variable modification, revealed a single high‐confidence glycopeptide, TYYLMDPSGN(Ng)AHK (peptide score = 43; mass error = −0.69 ppm), localizing the modification to the N531 site of the PDK1 protein(Figure 6B; Table S12). The mass difference between y^4^ and y^3^ ions (117.03 Da) matched the theoretical value for asparagine (Asn) + ^18^O deamidation, confirming that N531 is an N‐glycosylation site.

To further investigate the functional importance of N531 glycosylation in PDK1 protein, we generated a point mutant in which Asn531 (HA‐PDK1^WT^) was substituted with alanine (HA‐PDK1^N531A^) (Figure 6C). We first assessed the stability of the mutant protein using CHX chase assays. In both C4‐2 and LNCaP cells, the N531A mutant displayed a markedly shorter half‐life than wild‐type PDK1 following CHX treatment, indicating that the N531 residue plays a critical role in maintaining PDK1 protein stability (Figure 6D,E). Moreover, rescue experiments using the clone formation assay and TUNEL staining demonstrated that reintroducing HA‐PDK1^WT^ effectively restored the reduced proliferation and increased apoptosis caused by B4GALNT4 knockdown in C4‐2 cells. In contrast, HA‐PDK1^N531A^ failed to rescue these phenotypes, indicating that the N531 residue is essential for PDK1 function. (Figure 6F,G).

Integrated biochemical (global glycosylation reduction upon knockdown), MS‐based site mapping (N531 identification), and functional (N531A mutant) evidence provide strong support for a model in which B4GALNT4 mediates N‐glycosylation at N531 to stabilize PDK1, thereby activating the PI3K–AKT pathway and promoting PCa progression. Conversely, knockdown of B4GALNT4 accelerates PDK1 degradation and consequently suppresses PI3K–AKT pathway (Figure 6H).

### 2.7 Functional Assessment of B4GALNT4 in Mouse Models

**FIGURE 7:**
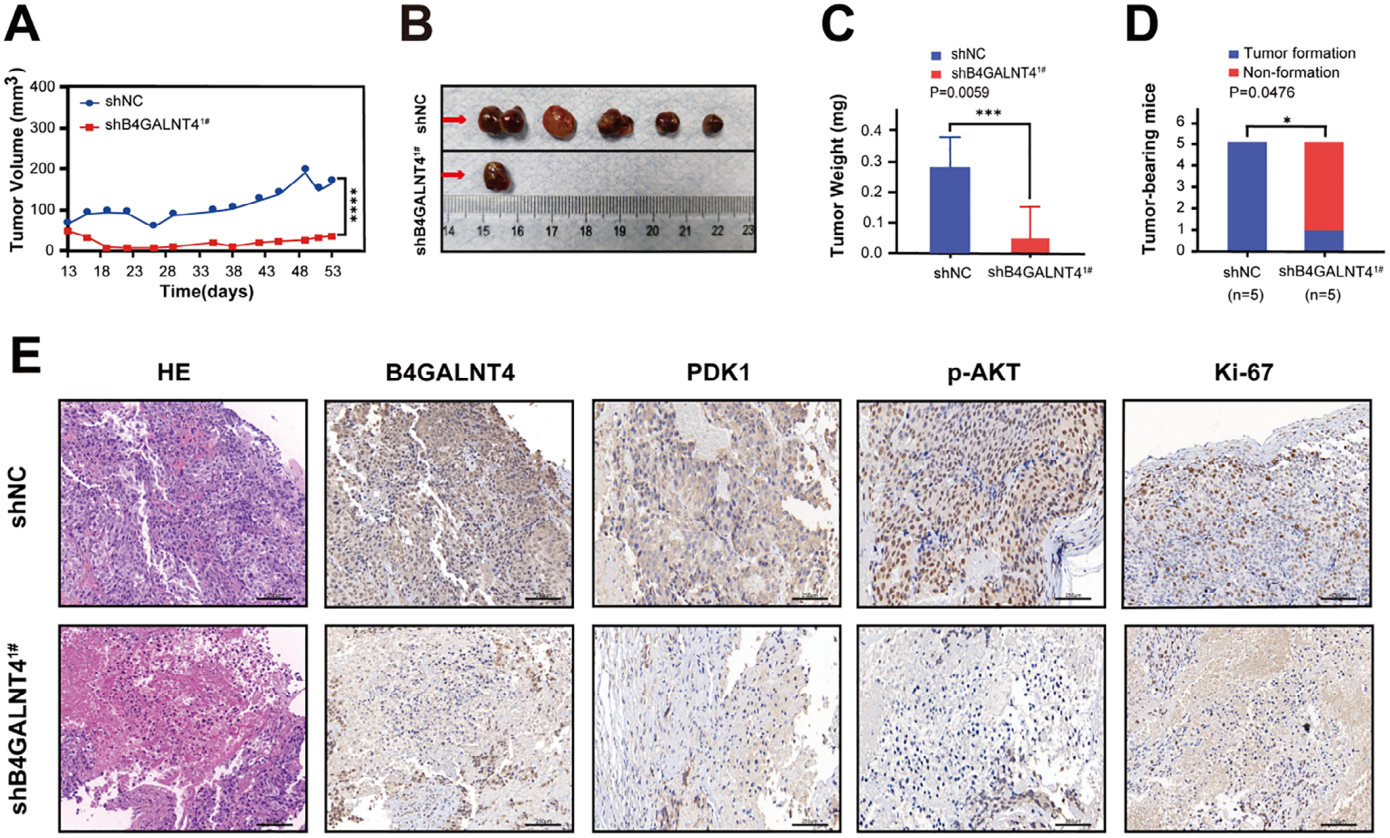
Functional assessment of B4GALNT4 in mouse models. (A) Tumor growth curves of nude mice subcutaneously transplanted with C4‐2 cells transfected with either shB4GALNT4^1#^ or shNC plasmid. (B) Representative images of tumors at the end of the experiment (53 days), showing reduced tumor size in the shB4GALNT4^1#^ group compared to the shNC group. (C) Tumor weight at the end of the experiment (53 days), with the shB4GALNT4^1#^ group showing significantly reduced tumor weight compared to the shNC group. (D) The number of nude mice with tumors was counted on the 53rd day of the experiment (C4‐2‐shNC group vs. C4‐2‐shB4GALNT4^1#^ group). Blue and red represent the number of tumors formed and non‐formed nude mice, respectively. (E) HE staining and IHC staining of B4GALNT4, PDK1, p‐AKT, and Ki‐67 proteins from tumor tissues of nude mice subcutaneously transplanted with shB4GALNT4^1#^ or shNC‐transfected C4‐2 cells (scale bar: 250 µm).

To evaluate the clinical translational potential of B4GALNT4, we investigated its function in a mouse xenograft model. We first established C4‐2 cells with stable B4GALNT4 knockdown (shB4GALNT4^1#^) and used them for xenograft tumor formation in nude mice. The results showed that, compared to the control group (shNC), B4GALNT4 knockdown significantly inhibited tumor growth (Figure 7A), with tumor volume being markedly reduced throughout the monitoring period (Figure 7B), and the final tumor masses being smaller and weighing significantly less (Figure 7C). Furthermore, the tumor formation rate was also significantly lower in the B4GALNT4 knockdown group (Figure 7D). HE staining revealed a decrease in cell density and a more loosely organized structure in the tumors from the B4GALNT4 knockdown group. IHC results indicated that B4GALNT4 downregulation led to a significant reduction in PDK1 and p‐AKT expression, while the proportion of Ki‐67‐positive cells was also reduced, suggesting a suppression of proliferation (Figure 7E). In summary, these findings demonstrate that B4GALNT4 plays a promoting role in the in vivo growth of PCa, and its downregulation can inhibit the PI3K‐PDK1‐AKT signaling pathway and impair tumor cell proliferation.

## 3 Discussion

This study has identified B4GALNT4 as a key molecule driving the malignant progression of high‐risk PCa. The significant overexpression of B4GALNT4 serves as a key biomarker for disease invasiveness and poor prognosis. Moreover, we have discovered for the first time that B4GALNT4 directly catalyzes N‐glycosylation at the N531 site of the PDK1 protein. This modification stabilizes PDK1 expression and continuously activates the PI3K‐AKT signaling pathway, thereby promoting the malignant progression of PCa. This important discovery fills a critical gap in PDK1 glycosylation research, establishing B4GALNT4 as the key link between aberrant glycosylation, persistent activation of oncogenic signaling, and the malignant clinical phenotype of PCa. It further highlights its driving role in PCa progression and fully demonstrates its significant value as a potential therapeutic target.

B4GALNT4, a key member of the B4GALNT glycosyltransferase family, plays a critical role in several biological processes linked to cancer progression. Recent studies suggest that B4GALNT4 may serve as a predictive biomarker for lymph node metastasis in obese patients with endometrial cancer [30]. In hepatocellular carcinoma, B4GALNT4 regulates microtubule spindle assembly by modulating the expression of PLK1 and RHAMM [16]. Additionally, several studies have confirmed that overexpression of B4GALNT4 significantly enhances the malignant phenotype of esophageal and ovarian cancers and is strongly associated with disease recurrence and poor prognosis [15, 31]. In this study, we also found that high expression of B4GALNT4 is a significant marker of malignant progression in PCa, holding important clinical application potential. As an effective risk stratification factor, it can accurately distinguish between high‐risk and non‐high‐risk PCa patients, providing valuable information to guide clinical decision‐making. Specifically, measuring B4GALNT4 levels in tumor tissues may assist existing clinical indicators (such as Gleason score, PSA, and imaging) in earlier and more accurate identification of patients with high progression risk, poor prognosis, and urgent need for active intervention. The strong association between B4GALNT4 overexpression and poor patient outcomes makes it a valuable independent prognostic biomarker, which can help optimize patient management. More importantly, B4GALNT4 can regulate PDK1 through glycosylation, thereby activating the PI3K‐AKT signaling pathway and promoting the malignant progression of PCa.

The PI3K‐AKT signaling pathway is a central regulatory network in PCa progression, with its aberrant activation closely linked to malignant phenotypes such as proliferation, invasion, migration, and treatment resistance [20]. This pathway enhances aerobic glycolysis (Warburg effect) by phosphorylating various nutrient transport proteins and metabolic enzymes, inhibiting apoptosis, and promoting PCa cell proliferation [32]. Studies have shown that the androgen receptor (AR) pathway and the PI3K‐AKT pathway have a negative feedback mechanism; combining inhibitors targeting these two pathways effectively reduces drug resistance [33]. Additionally, activation of the PI3K‐AKT pathway promotes the epithelial‐mesenchymal transition (EMT), driving the metastatic spread of PCa cells [34]. Our study further reveals that B4GALNT4 may activate the PI3K‐AKT signaling pathway by glycosylating PDK1, highlighting a previously unrecognized glycosylation‐dependent regulatory mechanism within this critical pathway.

As a key kinase in the PI3K‐AKT pathway, PDK1 plays a crucial role in the progression of various cancers by activating this pathway and subsequently regulating the downstream signaling molecules [20]. This role is also prominent in PCa [35, 36]. PDK1 then phosphorylates the Thr308 site of AKT, which is essential for full activation of AKT [26]. Additionally, various upstream signals (e.g., MAPK4, KDM4C) further enhance the PI3K‐AKT signaling pathway by increasing PDK1 protein expression, thereby promoting PCa progression [37, 38]. Furthermore, PDK1‐mediated activation of the PI3K‐AKT pathway directly drives downstream effectors such as mTORC1 and FOXO, promoting the proliferation, invasion, and migration of PCa cells [39, 40]. This study reveals for the first time a novel regulatory mode: We successfully identified N531 of PDK1 as the main glycosylation site, and confirmed that glycosylation of this site plays a critical role in maintaining the stability of PDK1. Unlike traditional PDK1 regulation, B4GALNT4 does not affect PDK1 transcription but directly enhances its protein stability via N‐glycosylation on the N531 site. Overall, B4GALNT4‐mediated glycosylation of PDK1 at the N531 site maintains the stability of the PDK1 protein and prolongs its half‐life, thereby facilitating sustained activation of the PI3K‐AKT pathway.

Based on these findings, B4GALNT4 is expected to be a novel therapeutic target and prognostic marker for PCa. Specifically, targeting B4GALNT4‐mediated glycosylation of PDK1 at the N531 residue could be a promising therapeutic strategy. Given the progress in PDK1 inhibitor development (e.g., BX‐795) [41], our study supports the therapeutic option of combining B4GALNT4 inhibitors with existing therapies (e.g., PDK1 inhibitors or ADT). Moreover, the high specificity of PDK1 glycosylation makes it an ideal target for precision drug design.

While this study comprehensively reveals the functional mechanism of B4GALNT4 in regulating PDK1 through glycosylation, some limitations remain. First, clinical validation using a larger external cohort is required. Second, our study focused on the N531 site of PDK1, but B4GALNT4 may also regulate other key signaling molecules, which warrants further exploration. Furthermore, further resolution of B4GALNT4's structural properties will aid in developing efficient and specific small‐molecule inhibitors.

## 4 Conclusion

Overall, this study reveals the molecular mechanism through which B4GALNT4 stabilizes PDK1 via glycosylation at the N531 site, activating the PI3K‐AKT signaling pathway to promote the malignant progression of PCa. B4GALNT4 not only serves as an important biomarker for high‐risk PCa, but also serves as a potential therapeutic target. The future development of therapeutic strategies targeting the B4GALNT4‐PDK1‐AKT regulatory axis is expected to significantly improve the prognosis of PCa patients.

## 5 Experimental Section

### 5.1 Identification and Bioinformatics Analysis of Glycogenes Associated With Prostate Cancer

Human glycogene information was extracted from the Glycogene Database (GGDB, https://acgg.asia/ggdb2/). RNA sequencing and clinical data for PCa patients were obtained from the CPGEA database [42], which we established previously, and the public TCGA database (TCGA‐PCa, https://portal.gdc.cancer.gov/). RNA sequencing data from the Second Military Medical University (SMMU, Eur Urol) [43] and the German Cancer Research Center (*Deutsches Krebsforschungszentrum* [DKFZ], Cancer Cell) [44] were additionally acquired from the cBioPortal (https://www.cbioportal.org/). Table S13 provides details on the downloaded databases.

Gene expression values from CPGEA (mRNA; fragments per kilobase of exon model per million reads mapped [FPKM]) were transformed to log(1+FPKM) and normalized using a Z‐score (mean‐centered). This normalization method was applied similarly to the TCGA‐PCa, SMMU, and DKFZ databases. The mean expression value was used for genes with multiple probes. Heatmaps of differentially expressed genes were generated using the “ComplexHeatmap” R package (Version 4.2.3). Unsupervised hierarchical clustering was performed with the Ward sum‐of‐squares method on data from the TCGA and CPGEA databases.

Differential gene analysis of log(1+FPKM)‐transformed data from TCGA and CPGEA was conducted using the “Limma R” package (version 4.16.0). Glycogenes associated with PCa were identified by comparing expression differences between tumor and normal tissues (|log fold change [logFC]| >1, adjusted *p*‐value <0.05) and between high‐risk and non‐high‐risk patients (|logFC| >0.5, adjusted *p*‐value <0.05). Univariate and multivariate logistic regression analyses were performed to assess the clinical correlation and prognostic significance of glycogenes in PCa. Cox survival analyses were conducted using the CPGEA and TCGA‐PCa database, and results were visualized with forest plots.

### 5.2 Patient Samples and Data Collection

Tissue sample collection was approved by the Ethics Committee of Changhai Hospital, Shanghai, China (approval number: CHEC2019‐012). Samples were collected according to standardized protocols in the hospital's Pathology Department, with informed consent obtained from participants or their relatives. PCa tissue samples were obtained from patients undergoing radical prostatectomy at Changhai Hospital. Clinical and follow‐up data were extracted from the hospital information system and the Prostate Cancer Follow‐Up Database (PC‐Follow).

### 5.3 Hematoxylin and Eosin (HE) Staining

PCa tissue slides were prepared by formalin fixation, paraffin embedding, and HE staining (G1004; Servicebio), following standardized protocols.

### 5.4 Immunohistochemistry (IHC)

Paraffin‐embedded tissue slides underwent gradient hydration and antigen retrieval. After blocking with goat serum (AR0009; BOSTER), slides were incubated overnight at 4°C with primary antibodies: B4GALNT4 (orb546266; Biorbyt), PDK1 (sc‐17765; Santa Cruz), Wisteria Floribunda Lectin (WFA) (B‐1355‐2; Vector Laboratories), phosphorylated AKT (p‐AKT) (sc‐514032; Santa Cruz), and Ki‐67 (A20018; Abclonal). Secondary antibodies were applied, followed by staining with 3,3'‐diaminobenzidine (DAB) (G1212‐200T; Servicebio).

### 5.5 Cell Culture

Human cell lines were maintained in the Laboratory of the Department of Urology, Changhai Hospital: BPH‐1 (benign prostate), C4‐2, LNCaP, DU145, PC3M, PC3 (PCa), and 293T cells. Prostate cell lines were cultured in RPMI1640 medium (Gibco), and 293T cells were cultured in Dulbecco's modified Eagle's medium (DMEM; Gibco), both supplemented with 10% fetal bovine serum and 1% penicillin/streptomycin. Cells were incubated at 37°C in 5% CO_2_.

### 5.6 Plasmid Construction and Mutation

The pLK0.1‐puro vector was modified with two homologous B4GALNT4‐shRNA sequences, resulting in pLK0.1‐puro‐shB4GALNT4^1#^ and pLK0.1‐puro‐shB4GALNT4^2#^ plasmids, with pLK0.1‐puro‐shNC used as a control. The PDK1 cDNA sequence tagged with hemagglutinin (HA) was cloned into the pcDNA3.1 plasmid to create pcDNA3.1(+)‐HA‐PDK1. The empty pcDNA3.1 vector served as a control. The HA‐PDK1^N531A^ mutant was generated using the Rapid Targeted Mutagenesis Kit (KM101; TianGen). All plasmids were sequence‐verified. Primer sequences are provided in Table S14.

### 5.7 Cell Line Transfection

Stable shB4GALNT4‐transfected cell lines were constructed by co‐transfecting plasmids containing B4GALNT4‐shRNA (pLK0.1‐puro‐shB4GALNT4^1#^, pLK0.1‐puro‐shB4GALNT4^2#^) or a control plasmid with envelope plasmids (PPAX and PMD2) into 293T cells using polyethylenimine (PEI) reagent. After 48 h, lentiviruses containing the target plasmids were collected and filtered. Stable cell lines were selected using puromycin (10 µg/mL).

For transient PDK1 overexpression, 293T or PCa cells were transfected with pcDNA3.1(+)‐HA‐PDK1 or the empty vector using PEI reagent for 8 h. Transfection efficiency was confirmed by western blotting and quantitative reverse transcription‐PCR (qRT‐PCR).

### 5.8 RNA Extraction and qRT‐PCR

RNA was extracted using a Cell/Tissue RNA Extraction Kit (RC101; Vazyme). Reverse transcription was performed using HiScriptIII RT SuperMix (RTQ‐201; TOROIVD). Quantitative PCR was performed using QuantStudio 6 Flex (Applied Biosystems) with SYBR Green qPCR Mix (QST‐100; TOROIVD). Primers are listed in Table S14, with triplicate reactions for each sample.

### 5.9 Western Blotting

Cell lysates were prepared using RIPA buffer (PC102; Epizyme) and PMSF (G2008‐1ML; Servicebio). Protein concentration was measured using the BCA Protein Assay Kit (ZJ102; Epizyme). Proteins were separated by SDS‐PAGE (10%) and transferred onto PVDF membranes (WJ002; Epizyme). Membranes were blocked with 5% skimmed milk and incubated overnight with primary antibodies. After washing, secondary antibodies were applied, and signal detection was performed with the Enhanced Chemiluminescence Kit (SQ101; Epizyme).

### 5.10 Lectin Histochemistry and Lectin Blotting

WFA lectin histochemistry was performed to examine the association between B4GALNT4 expression and GalNAc glycosylation in PCa tissues. After deparaffinization, antigen retrieval, and blocking with 5% BSA, the sections were incubated overnight at 4°C with biotinylated WFA (Vector Laboratories). Bound lectins were detected using streptavidin–HRP and visualized with DAB, followed by hematoxylin counterstaining.

To assess global WFA‐reactive glycoproteins in cells, lectin blotting was conducted. Total proteins from C4‐2 and LNCaP cells were separated by SDS–PAGE and transferred to PVDF membranes. After blocking, the membranes were incubated with biotinylated WFA, followed by anti‐biotin antibody or streptavidin–HRP. Signals were detected using ECL. Equal loading was verified by silver staining of the transferred membranes following the manufacturer's instructions. The detailed procedures are provided in the Supplementary Methods.

### 5.11 Immunofluorescence

Cells were fixed in 4% paraformaldehyde (G1101; Servicebio) and permeabilized in 0.5% Triton X‐100 (GC204003; Servicebio). Blocking was done with 5% BSA. Cells were incubated with biotinylated WFA lectin overnight at 4°C, followed by FITC‐conjugated avidin (bsF‐0312P; Bioss) for 1 h. Nuclei were stained with DAPI (C1006; Beyotime), and fluorescent cells were observed under a fluorescence microscope (DM5000B; Leica).

### 5.12 Cell Proliferation and Activity Assays

Tumor cells (1 × 10^3^ cells/well) were seeded in 96‐well plates and incubated overnight. Cell proliferation was measured using the CCK‐8 assay (G4103; Servicebio) at 1, 2, 3, 4, and 5 days. At each time point, 10 µL of CCK‐8 reagent was added, and cells were incubated for 2 h at 37°C. Absorbance at 490 nm was measured using a microplate reader (SpectraMax Paradigm; Molecular Devices).

### 5.13 Clone Formation Assay

Tumor cells were seeded in six‐well plates and incubated for 2 weeks. After fixation with paraformaldehyde (G1101; Servicebio), cells were stained with crystal violet (G1014; Servicebio). Clones were quantified using ImageJ software.

### 5.14 Real‐Time Live‐Cell Dynamic Imaging Assay

Tumor cells (1 × 10^3^ cells/well) were seeded in 24‐well plates and incubated at 37°C until adhesion. Cell proliferation was monitored using the zenCELL owl device (InnoME GmbH) and analyzed with zenCELL software (Version 3.4, InnoME GmbH) to generate images, data, videos, and curves.

### 5.15 TdT‐dUTP Nick End‐Labeling (TUNEL) Apoptosis Assay

Tumor cells were fixed with 4% paraformaldehyde (G1101; Servicebio) for 30 min, washed with PBS containing 0.3% Triton X‐100 (GC204003; Servicebio), and incubated with 50 µL TUNEL assay solution (TdT enzyme and fluorescent labeling) for 1 h at 37°C. After staining with DAPI (C1006; Beyotime), TUNEL‐positive cells were visualized under a fluorescence microscope (DM5000B; Leica), photographed, and analyzed.

### 5.16 Wound‐Healing Assays

Cells were seeded in six‐well plates (5 × 10^5^ cells/well). Wounds were created by scraping the cell monolayer using a 200 µL pipette tip, followed by gentle washing with PBS and incubation in serum‐free medium. An inverted microscope was used to observe and record wound closure, and images were captured at 24, 48, and 72 h.

### 5.17 Transwell Assays

Transwell chambers (353091; Corning Inc.) were coated with 100 µL Matrigel (354234; Corning Inc.) and incubated for 6 h. Tumor cells (1 × 10^5^) in serum‐free medium were added to the upper chamber, and 600 µL medium with 30% FBS was added to the lower chamber. After 24 or 48 h incubation at 37°C, cells were fixed with 4% paraformaldehyde (G1101; Servicebio), stained with crystal violet (G1014; Servicebio), and counted. Data are shown as mean±SD from three independent experiments.

### 5.18 RNA Sequencing (RNA‐Seq) and Differential Expression Analysis

Total RNA was extracted from C4‐2‐shNC and C4‐2‐shB4GALNT4^1#^ cells using the RNA Extraction Kit (RC101; Vazyme). cDNA libraries were constructed through PCR enrichment after assessing RNA quality, concentration, and integrity. Libraries were sequenced on the Illumina NovaSeq 6000 platform. Raw FASTQ data were quality controlled to generate clean data, which were aligned to the reference genome using Hisat2 v2.0.4. FPKM values were calculated with StringTie. Differential gene expression was analyzed using DESeq2 and edgeR (log2FC≥2, FDR<0.01). KEGG enrichment analysis was performed using the “clusterProfiler” R package (version 4.16.0).

### 5.19 Co‐Immunoprecipitation (Co‐IP)

Cells were lysed in pre‐cooled PBS with IP cell lysate (YJ204; Epizyme) on ice for 30 min. Lysates were centrifuged at 12 000 rpm for 30 min at 4°C. Supernatants were incubated overnight at 4°C with primary antibodies (B4GALNT4, PDK1, HA‐Tag, Flag‐Tag). Protein A/G Agarose microspheres (sc‐2003; Santa Cruz Biotechnology) were added, and the mixture was incubated for 2 h at 4°C. After washing and centrifugation, the precipitate was resuspended in 1× loading buffer (G2013‐1ML; Servicebio), boiled for 10 min, and analyzed by western blotting.

### 5.20 Cycloheximide Chase Assay

Tumor cells were seeded in six‐well plates (5 × 10^5^ cells/well) and treated with 50 µg/mL cycloheximide (CHX) for 0, 2, 4, 6, 8, and 10 h. Lysates were analyzed by SDS‐PAGE and western blotting using antibodies against B4GALNT4 and PDK1. Protein decay was plotted, and data were expressed as mean ± SD from three independent experiments.

### 5.21 Protein Degradation Assays

Cells were seeded in six‐well plates (5 × 10^5^ cells/well) and treated with chloroquine (CQ, 25 µm) or MG132 (10 µm) for 6 h. Control cells were treated with Dimethyl Sulfoxide (DMSO). Cell lysates were analyzed by western blotting to evaluate protein degradation.

### 5.22 4D Label‐Free Quantitative Proteomic Analysis

To investigate the proteomic alterations induced by B4GALNT4 knockdown, we conducted a label‐free quantitative analysis on C4‐2 cells stably expressing shNC or shB4GALNT4. Total proteins were extracted, reduced, alkylated, and digested with trypsin. The resulting peptides were desalted and analyzed by liquid chromatography‐tandem mass spectrometry (LC‐MS/MS) using an Orbitrap Fusion Tribrid mass spectrometer (Thermo Scientific) operating in data‐dependent acquisition mode.

MS data were searched against the UniProt Human database (v20210721) using MaxQuant (v1.6.15.0) with standard settings. Label‐free quantification (LFQ) was performed with the MaxLFQ algorithm, and the ‘match between runs’ feature was enabled to enhance quantification accuracy. Proteins were considered differentially expressed if they exhibited a |fold change| > 1.5 with an associated *p*‐value < 0.05 (Student's t‐test). Subsequently, KEGG pathway enrichment analysis was performed on the DEPs using the clusterProfiler package in R. A comprehensive description of all reagents, LC gradients, MS instrumentation parameters, and database search criteria is available in the Supplementary methods.

### 5.23 Prediction of PDK1 Glycosylation Sites

Bioinformatic prediction of glycosylation sites: N‐ and O‐glycosylation sites on PDK1 were predicted using the NetNGlyc‐1.0 (https://services.healthtech.dtu.dk/services/NetNGlyc‐1.0/) and NetOGlyc‐4.0 (https://services.healthtech.dtu.dk/services/NetOGlyc‐4.0/) servers.

### 5.24 Identification of PDK1 Glycosylation Sites by PNGase F–Assisted LC–MS/MS

To identify the glycosylation site(s) on PDK1, C4‐2 cell lysates were subjected to immunoprecipitation using a PDK1‐specific antibody. The immunocomplexes were resolved by SDS–PAGE, and the PDK1 band was excised for in‐gel digestion. Prior to proteolysis, gel slices were incubated with PNGase F overnight at 37°C in an ^18^O‐labeled ammonium bicarbonate buffer to remove N‐linked glycans. This treatment converts glycosylated Asn to Asp, generating a characteristic +2.99 Da mass shift that enables precise identification of N‐glycosylation sites.

Peptides obtained after reduction, alkylation, and trypsin digestion were analyzed using an EASY‐nLC 1200 system coupled to a Q Exactive HF‐X mass spectrometer. MS data were acquired in DDA mode, and raw files were processed using Proteome Discoverer 2.4. Searches were performed against the human PDK1 sequence with Trypsin/P specificity, fixed carbamidomethylation (Cys), and variable oxidation (Met), N‐terminal acetylation, and ^18^O‐deamidation (Asn) to detect PNGase F–induced deglycosylation. High‐confidence peptide matches were retained based on standard scoring criteria.

Detailed experimental procedures for immunoprecipitation, in‐gel digestion, and LC–MS/MS analysis are provided in the Supporting Methods.

### 5.25 Animal Experiments

Animal experiments were approved by the Ethics Committee of Union Hospital, Fujian Medical University (approval number: IACUC FJMU 2023‐0030). Ten male BALB/C nude mice (3‐4 weeks old) were randomly assigned to C4‐2‐shB4GALNT4^1#^ (n = 5) and C4‐2‐shNC (n = 5) groups. Tumor xenografts were established by subcutaneously injecting 3 × 10^6^ tumor cells in Matrigel. Tumor growth was monitored every 3 days, and euthanasia was performed if tumors exceeded 1000 mm^3^ or mice became cachectic. Tumor weight and IHC analysis were performed.

### 5.26 Statistical Analysis

Statistical analyses were conducted using GraphPad Prism. Mann–Whitney U‐test and *t*‐test were used for non‐normally and normally distributed data, respectively. Chi‐square tests were used for enumeration data. Table S14 contains the sequences of primers and oligonucleotides used in this study. All other data are available from the corresponding author upon reasonable request.

## Author Contributions

**Shaoqin Jiang**: Writing – original draft, Methodology, Investigation, Conceptualization. **Min Qu**: Methodology, Conceptualization. **Yan Wang**: Methodology, Investigation. **Shaoshan Lin**: Methodology, Investigation. **Qingfu Su**, Writing – review & editing, Project administration, Conceptualization. **Jili Zhang**: Resources. **Xiaochen Sun**: Methodology. **Haolan Yu**: Investigation. **Wei Bao**: Methodology. **Mengqiang Li**: Writing – review & editing. **Ruiling Dou**: Writing – review & editing, Project administration, Conceptualization. **Chenghua Yang**: Writing – review & editing, Supervision, Conceptualization. **Xu Gao**: Writing – review & editing, Supervision, Conceptualization.

## Funding

This work was supported by funds from the Fujian Natural Sciences Foundation (Grant number: 2022J01260 to M.L.), the National Science Foundation of China (Grant numbers: 82203309 to S.J., 81772695, 82373011 to C.Y., and 82272793 to X.G.), and Joint Funds for the innovation of Science and Technology, Fujian province (Grant numbers: 2025Y9291 to S.J.).

## Conflicts of Interest

The authors declare no conflict of interest.

## Supporting information




**Supporting File 1**: advs74410‐sup‐0001‐SuppMat.docx.

**Supporting File 2**: advs74410‐sup‐0002‐TableS8.xlsx.

**Supporting File 3**: advs74410‐sup‐0003‐TableS9.xlsx.

## Data Availability

Raw RNA‐sequencing data are available in the Open Archive for Miscellaneous Data (OMIX) under accession number OMIX012180 (https://ngdc.cncb.ac.cn/omix/releaseList, BioProject: PRJCA047481). Raw proteomic mass‐spectrometry data and glycoproteomics data have been deposited in the iProX repository and assigned ProteomeXchange dataset identifier PXD069701 (https://proteomecentral.proteomexchange.org/ui?pxid = PXD069701, iProX project ID IPX0013375000; sub‐project IDs IPX0013375003 for proteomics and IPX0013375004 for glycoproteomics).

## References

[1] R. L. Siegel , A. N. Giaquinto , and A. Jemal , “Cancer Statistics,” CA: a Cancer Journal for Clinicians 74, no. 1 (2024): 12–49, 10.3322/caac.21820. 38230766

[2] S. Sandhu , C. M. Moore , E. Chiong , H. Beltran , R. G. Bristow , and S. G. Williams , “Prostate Cancer,” The Lancet 398, no. 10305 (2021): 1075–1090, 10.1016/S0140-6736(21)00950-8. 34370973

[3] Y. He , W. Xu , Y. Xiao , H. Huang , D. Gu , and S. Ren , “Targeting Signaling Pathways in Prostate Cancer: Mechanisms and Clinical Trials,” Signal Transduction and Targeted Therapy 7, no. 1 (2022): 198, 10.1038/s41392-022-01042-7. 35750683 PMC9232569

[4] S. Chowdhury , A. Bjartell , N. Lumen , et al., “Real‐World Outcomes in First‐Line Treatment of Metastatic Castration‐Resistant Prostate Cancer: The Prostate Cancer Registry,” Targeted Oncology 15, no. 3 (2020): 301–315, 10.1007/s11523-020-00720-2. 32500294 PMC7283204

[5] M. He , X. Zhou , and X. Wang , “Glycosylation: Mechanisms, Biological Functions and Clinical Implications,” Signal Transduction and Targeted Therapy 9, no. 1 (2024): 194, 10.1038/s41392-024-01886-1. 39098853 PMC11298558

[6] X. Xu , Q. Peng , X. Jiang , et al., “Altered Glycosylation in Cancer: Molecular Functions and Therapeutic Potential,” Cancer Communications 44, no. 11 (2024): 1316–1336, 10.1002/cac2.12610. 39305520 PMC11570773

[7] S. S. Pinho and C. A. Reis , “Glycosylation in Cancer: Mechanisms and Clinical Implications,” Nature Reviews Cancer 15, no. 9 (2015): 540–555, 10.1038/nrc3982. 26289314

[8] Y. Chen , L. Su , C. Huang , et al., “Galactosyltransferase B4GALT1 Confers Chemoresistance in Pancreatic Ductal Adenocarcinomas by Upregulating N‐linked Glycosylation of CDK11p110,” Cancer Letters 500(2021): 228–243, 10.1016/j.canlet.2020.12.006. 33309857

[9] Y. Cui , J. Li , P. Zhang , et al., “B4GALT1 promotes Immune Escape by Regulating the Expression of PD‐L1 at Multiple Levels in Lung Adenocarcinoma,” Journal of Experimental & Clinical Cancer Research 42, no. 1 (2023): 146, 10.1186/s13046-023-02711-3. 37303063 PMC10259029

[10] Q. Zhu , H. Wang , S. Chai , et al., “O‐GlcNAcylation Promotes Tumor Immune Evasion by Inhibiting PD‐L1 Lysosomal Degradation,” Proceedings of the National Academy of Sciences 120, no. 13 (2023): 2216796120, 10.1073/pnas.2216796120. PMC10068856 36943877

[11] T. Zhao , L. Jia , J. Li , et al., “Heterogeneities of Site‐Specific N‐Glycosylation in HCC Tumors with Low and High AFP Concentrations,” Frontiers in Oncology 10(2020): 496, 10.3389/fonc.2020.00496. 32426269 PMC7212448

[12] M. Zhang , S. Cheng , Y. Jin , Y. Zhao , and Y. Wang , “Roles of CA125 in Diagnosis, Prediction, and Oncogenesis of Ovarian Cancer,” Biochimica et Biophysica Acta (BBA)—Reviews on Cancer 1875, no. 2 (2021): 188503, 10.1016/j.bbcan.2021.188503. 33421585

[13] A. Silsirivanit , “Glycosylation Markers in Cancer,” Advances in Clinical Chemistry 89(2019): 189–213, 10.1016/bs.acc.2018.12.005. 30797469

[14] W. Tang , M. Li , X. Qi , et al., “β1,4‐Galactosyltransferase V Modulates Breast Cancer Stem Cells through Wnt/β‐catenin Signaling Pathway,” Cancer Research and Treatment 52, no. 4 (2020): 1084–1102, 10.4143/crt.2020.093. 32599982 PMC7577798

[15] M. Anugraham , F. Jacob , A. V. Everest‐Dass , et al., “Tissue Glycomics Distinguish Tumour Sites in Women with Advanced Serous Adenocarcinoma,” Molecular Oncology 11, no. 11 (2017): 1595–1615, 10.1002/1878-0261.12134. 28853212 PMC5663998

[16] Z. Dai , K. Wang , and Y. Gao , “The Critical Role of B4GALT4 in Promoting Microtubule Spindle Assembly in HCC through the Regulation of PLK1 and RHAMM Expression,” Journal of Cellular Physiology 237, no. 1 (2022): 617–636, 10.1002/jcp.30531. 34270095

[17] J. Zhang , S. Jiang , D. Gu , et al., “Identification of Novel Molecular Subtypes and a Signature to Predict Prognosis and Therapeutic Response Based on Cuproptosis‐related Genes in Prostate Cancer,” Frontiers in Oncology 13(2023): 1162653, 10.3389/fonc.2023.1162653. 37205181 PMC10185853

[18] K. Hirano and K. Furukawa , “Biosynthesis and Biological Significances of LacdiNAc Group on N‐ and O‐Glycans in Human Cancer Cells,” Biomolecules 12, no. 2 (2022): 195, 10.3390/biom12020195. 35204696 PMC8961560

[19] Y. Haga , M. Uemura , S. Baba , et al., “Identification of Multisialylated LacdiNAc Structures as Highly Prostate Cancer Specific Glycan Signatures on PSA,” Analytical Chemistry 91, no. 3 (2019): 2247–2254, 10.1021/acs.analchem.8b04829. 30669833

[20] R. Wang , Z. Qu , Y. Lv , et al., “Important Roles of PI3K/AKT Signaling Pathway and Relevant Inhibitors in Prostate Cancer Progression,” Cancer Medicine 13, no. 21 (2024): 70354, 10.1002/cam4.70354. PMC11529649 39485722

[21] S. Yue , J. Li , S. Lee , et al., “Cholesteryl Ester Accumulation Induced by PTEN Loss and PI3K/AKT Activation Underlies human Prostate Cancer Aggressiveness,” Cell Metabolism 19, no. 3 (2014): 393–406, 10.1016/j.cmet.2014.01.019. 24606897 PMC3969850

[22] W. Zhang , Z. Liu , S. Wen , et al., “NEIL3 promotes the Carcinogenesis of Prostate Cancer by Activating PI3K/Akt/mTOR Signaling,” Hormones & Cancer 16, no. 1 (2025): 967, 10.1007/s12672-025-02625-w. PMC12125448 40447877

[23] S. El‐Naggar , Y. Liu , and D. C. Dean , “Mutation of the Rb1 Pathway Leads to Overexpression of mTor, Constitutive Phosphorylation of Akt on Serine 473, Resistance to Anoikis, and a Block in c‐Raf Activation,” Molecular and Cellular Biology 29, no. 21 (2009): 5710–5717, 10.1128/MCB.00197-09. 19703998 PMC2772742

[24] K. K. Velpula , A. Bhasin , S. Asuthkar , and A. J. Tsung , “Combined Targeting of PDK1 and EGFR Triggers Regression of Glioblastoma by Reversing the Warburg Effect,” Cancer Research 73, no. 24 (2013): 7277–7289, 10.1158/0008-5472.CAN-13-1868. 24148623

[25] D. Haas‐Kogan , N. Shalev , M. Wong , G. Mills , G. Yount , and D. Stokoe , “Protein Kinase B (PKB/Akt) Activity Is Elevated in Glioblastoma Cells due to Mutation of the Tumor Suppressor PTEN/MMAC,” Current Biology 8, no. 21 (1998): 1195–1198, 10.1016/s0960-9822(07)00493-9. 9799739

[26] M. Maurer , T. Su , L. H. Saal , et al., “3‐Phosphoinositide–Dependent Kinase 1 Potentiates Upstream Lesions on the Phosphatidylinositol 3‐Kinase Pathway in Breast Carcinoma,” Cancer Research 69, no. 15 (2009): 6299–6306, 10.1158/0008-5472.CAN-09-0820. 19602588 PMC2727605

[27] N. Mottet , R. C. van den Bergh , E. Briers , et al., “EAU‐EANM‐ESTRO‐ESUR‐SIOG Guidelines on Prostate Cancer—2020 Update. Part 1: Screening, Diagnosis, and Local Treatment with Curative Intent,” European Urology 79, no. 2 (2021): 243–262, 10.1016/j.eururo.2020.09.042. 33172724

[28] N. Aoki , T. Matsuda , T. Sakiyama , K. Iwatsuki , and K. Furukawa , “Species‐specific β‐N‐acetylgalactosaminylation of Serum IgG Proteins,” Biochimica et Biophysica Acta (BBA)—General Subjects 1334, no. 2–3 (1997): 207–213, 10.1016/s0304-4165(96)00094-3. 9101715

[29] O. Haji‐Ghassemi , M. Gilbert , J. Spence , et al., “Molecular Basis for Recognition of the Cancer Glycobiomarker, LacdiNAc (GalNAc[β1→4]GlcNAc), by Wisteria floribunda Agglutinin,” Journal of Biological Chemistry 291, no. 46 (2016): 24085–24095, 10.1074/jbc.M116.750463. 27601469 PMC5104934

[30] V. M. López‐Ozuna , L. Kogan , M. Y. Hachim , et al., “Identification of Predictive Biomarkers for Lymph Node Involvement in Obese Women with Endometrial Cancer,” Frontiers in Oncology 11(2021): 695404, 10.3389/fonc.2021.695404. 34307159 PMC8292832

[31] H. Baba , M. Kanda , Y. Sato , et al., “Expression and Malignant Potential of B4GALNT4 in Esophageal Squamous Cell Carcinoma,” Annals of Surgical Oncology 27, no. 9 (2020): 3247–3256, 10.1245/s10434-020-08431-8. 32253672

[32] F. Fontana , G. Giannitti , S. Marchesi , and P. Limonta , “The PI3K/Akt Pathway and Glucose Metabolism: A Dangerous Liaison in Cancer,” International journal of Biological Sciences 20, no. 8 (2024): 3113–3125, 10.7150/ijbs.89942. 38904014 PMC11186371

[33] B. Y. Shorning , M. S. Dass , M. J. Smalley , and H. B. Pearson , “The PI3K‐AKT‐mTOR Pathway and Prostate Cancer: At the Crossroads of AR, MAPK, and WNT Signaling,” International Journal of Molecular Sciences 21, no. 12 (2020): 4507, 10.3390/ijms21124507. 32630372 PMC7350257

[34] M. Hashemi , A. Taheriazam , P. Daneii , et al., “Targeting PI3K/Akt Signaling in Prostate Cancer Therapy,” Journal of Cell Communication and Signaling 17, no. 3 (2023): 423–443, 10.1007/s12079-022-00702-1. 36367667 PMC10409967

[35] T. Maehama , G. S. Taylor , and J. E. Dixon , “PTEN and Myotubularin: Novel Phosphoinositide Phosphatases,” Annual Review of Biochemistry 70(2001): 247–279, 10.1146/annurev.biochem.70.1.247. 11395408

[36] K. M. Hill , S. Kalifa , J. R. Das , et al., “The Role of PI 3‐Kinase p110β in AKT Signally, Cell Survival, and Proliferation in human Prostate Cancer Cells,” The Prostate 70, no. 7 (2010): 755–764, 10.1002/pros.21108. 20058239

[37] Y. Yan , T. Dai , M. Guo , et al., “A Review of Non‐classical MAPK family Member, MAPK4: A Pivotal Player in Cancer Development and Therapeutic Intervention,” International Journal of Biological Macromolecules 271, no. 2 (2024): 132686, 10.1016/j.ijbiomac.2024.132686. 38801852

[38] C. Lin , B. Wang , B. Chen , et al., “Histone Demethylase KDM4C Stimulates the Proliferation of Prostate Cancer Cells via Activation of AKT and c‐Myc,” Cancers 11, no. 11 (2019): 1785, 10.3390/cancers11111785. 31766290 PMC6896035

[39] U. K. Misra and S. V. Pizzo , “Activated α2‐macroglobulin Binding to Cell Surface GRP78 Induces T‐loop Phosphorylation of Akt1 by PDK1 in Association with Raptor,” PLoS ONE 9, no. 2 (2014): 88373, 10.1371/journal.pone.0088373. PMC3916429 24516643

[40] V. Modur , R. Nagarajan , B. M. Evers , and J. Milbrandt , “FOXO Proteins Regulate Tumor Necrosis Factor‐related Apoptosis Inducing Ligand Expression,” Journal of Biological Chemistry 277, no. 49 (2002): 47928–47937, 10.1074/jbc.M207509200. 12351634

[41] A. Su , M. Qiu , Y. Li , et al., “BX‐795 Inhibits HSV‐1 and HSV‐2 Replication by Blocking the JNK/p38 Pathways without Interfering with PDK1 Activity in Host Cells,” Acta Pharmacologica Sinica 38, no. 3 (2017): 402–414, 10.1038/aps.2016.160. 28112176 PMC5342671

[42] J. Li , C. Xu , H. J. Lee , et al., “A Genomic and Epigenomic Atlas of Prostate Cancer in Asian Populations,” Nature 580, no. 7801 (2020): 93–99, 10.1038/s41586-020-2135-x. 32238934

[43] S. Ren , G. Wei , D. Liu , et al., “Whole‐genome and Transcriptome Sequencing of Prostate Cancer Identify New Genetic Alterations Driving Disease Progression,” European Urology 73, no. 3 (2018): 322–339, 10.1016/j.eururo.2017.08.027. 28927585

[44] C. Gerhauser , F. Favero , T. Risch , et al., “Molecular Evolution of Early‐Onset Prostate Cancer Identifies Molecular Risk Markers and Clinical Trajectories,” Cancer Cell 34, no. 6 (2018): 996–1011, 10.1016/j.ccell.2018.10.016. 30537516 PMC7444093

