# Supplementary material for: B4GALNT4‐Mediated Glycosylation of PDK1 Activates the PI3K‐AKT Signaling Pathway to Promote Prostate Cancer Progression
Source: Adv Sci (Weinh). 2026 Feb 16;13(23):e11293. doi: 10.1002/advs.202511293 (PMC13104079; doi:10.1002/advs.202511293)
Supplement: Supplementary file 1 — Supporting File 1: advs74410‐sup‐0001‐SuppMat.docx. [file ADVS-13-e11293-s002.docx]

**Supplementary methods**

**5.10. Lectin histochemistry and Lectin blotting**

Wisteria floribunda agglutinin (WFA) is a plant lectin that specifically recognizes terminal N-acetylgalactosamine (GalNAc) residues on glycan chains and is widely used to detect both O- and N-linked GalNAc-containing glycoconjugates. To examine the relationship between B4GALNT4 expression and global GalNAc glycosylation in PCa tissues, WFA lectin histochemistry was performed. B4GALNT4 levels were first evaluated by immunohistochemistry (IHC), and tissue sections exhibiting high or low B4GALNT4 expression were selected for WFA histochemistry. After deparaffinization, rehydration, and antigen retrieval, endogenous peroxidase activity was quenched, and the sections were blocked with 5% bovine serum albumin (BSA). The slides were then incubated overnight at 4°C with biotinylated WFA (B-1355-2; Vector Laboratories) diluted in blocking buffer. After washing, bound lectins were visualized using a streptavidin–horseradish peroxidase (streptavidin-HRP) conjugate, followed by development with a DAB chromogenic substrate. Nuclei were counterstained with hematoxylin, and images were captured under a light microscope.

To assess global changes in WFA-reactive glycoproteins following B4GALNT4 knockdown in prostate cancer cell lines, lectin blotting was conducted. Total protein extracts from C4-2 and LNCaP cells were resolved by SDS–PAGE and transferred onto PVDF membranes (0.45 μm pore size; WJ002, Epizyme). The membranes were blocked with 5% BSA and incubated overnight at 4°C with biotinylated WFA diluted in blocking buffer. After washing, the membranes were incubated with an anti-biotin antibody (5571S; Cell Signaling Technology) for 1 hour at room temperature, or alternatively, an HRP-conjugated streptavidin was applied directly. WFA-bound proteins were visualized using an enhanced chemiluminescence (ECL) substrate (SQ101; Epizyme). To verify equal protein loading, the transferred membranes were subsequently subjected to silver staining using a Rapid Silver Staining Kit (P0017S; Beyotime), following the manufacturer’s recommended protocol.

**5.22. Four-dimensional label-free quantitative proteomic analysis**

To investigate the global protein expression profile upon B4GALNT4 knockdown, a label-free quantitative proteomics approach was employed. C4-2 cells stably expressing shNC or shB4GALNT4 were cultured in biological triplicates. Total protein was extracted from cell lysates using 200ml Radio Immunoprecipitation Assay (RIPA, NCM Biotech, WB3100) lysis buffer supplemented with 2ml protease inhibitor (Epizyme Biomedical, Shanghai, GRF101) and 2ml phosphatase inhibitor cocktail (GRF101). The protein concentration was determined using a Bicin-choninic Acid (BCA) assay kit (Epizyme Biomedical, Shanghai, ZJ102) according to the manufacturer's instructions.

For each sample, 100 μg of protein was subjected to reduction with 10 mM dithiothreitol (DTT) at 56°C for 30 min and alkylation with 55 mM iodoacetamide (IAA) at room temperature in the dark for 15 min. The proteins were then precipitated using cold acetone, reconstituted, and digested overnight at 37°C with sequencing-grade trypsin (NCM Biotech, C100C1) at an enzyme-to-protein ratio of 1:50. The resulting peptides were desalted using C18 StageTips (Thermo Scientific, 84850) and lyophilized.

The dried peptides were reconstituted in 0.1% formic acid and separated on an EASY-nLC 1200 UPLC system (Thermo Scientific) coupled to an Orbitrap Fusion Tribrid mass spectrometer. Peptides were loaded onto a homemade analytical column (15 cm length, 75 μm i.d.) packed with ReproSil-Pur C18-AQ resin (1.9 μm particle size; Dr. Maisch GmbH) and separated with a 90-min linear gradient: from 6% to 25% solvent B (0.1% formic acid in 80% acetonitrile) over 70 min, 25% to 35% in 12 min, climbing to 80% in 3 min, and holding at 80% for the last 5 min, at a constant flow rate of 450 nL/min. Solvent A was 0.1% formic acid in water.

The mass spectrometer was operated in data-dependent acquisition (DDA) mode. The full MS scan range was set to 350–1800 *m/z* with a resolution of 120,000. The automatic gain control (AGC) target was set to 4E5, and the maximum injection time was 50 ms. The most intense precursor ions with a charge state of 2-7 were selected for fragmentation by higher-energy collisional dissociation (HCD) with a normalized collision energy (NCE) set to 28%. LC–MS/MS spectra were acquired with a resolution of 30,000. The AGC target was set to 5E4, the maximum injection time was 54 ms, and the dynamic exclusion duration was set to 18 s. The instrument was set to cycle between MS1 and MS2 scans with a top-speed mode (3s cycle time).

The acquired raw data were processed using MaxQuant software (version 1.6.15.0) against the UniProt Human reference proteome database (Homo_sapiens_9606_SP_20210721.fasta, 20,387 sequences). The search parameters were set as follows: trypsin/P as the enzyme with up to two missed cleavages; carbamidomethylation on cysteine as a fixed modification; oxidation of methionine and protein N-terminal acetylation as variable modifications; precursor mass tolerance of 10 ppm; and fragment mass tolerance of 0.02 Da. The false discovery rate (FDR) was set to <1% at both the peptide-spectrum match (PSM) and protein levels. The ‘match between runs’ feature was enabled to transfer identifications across samples. Protein quantification was performed using the label-free quantification (LFQ) algorithm MaxLFQ integrated in MaxQuant. Proteins were required to have at least one unique peptide for identification.

Differentially expressed proteins (DEPs) were defined based on the following criteria: a fold change (shB4GALNT4/shNC) > 1.5 or < 1/1.5 and a Student's t-test p-value < 0.05. The DEPs were subsequently subjected to KEGG pathway enrichment analysis using the clusterProfiler R package.

**5.23. Prediction of PDK1 glycosylation sites**

N- and O-glycosylation sites on the PDK1 protein were predicted using the NetNGlyc-1.0 (https://services.healthtech.dtu.dk/services/NetNGlyc-1.0/) and NetOGlyc-4.0 (https://services.healthtech.dtu.dk/services/NetOGlyc-4.0/) servers. The canonical amino acid sequence of human PDK1 in FASTA format was submitted to the online tools. Predictions were performed using default parameters. NetNGlyc-1.0 predicts N-glycosylation sites based on the presence of the Asn-X-Ser/Thr motif (where X is any amino acid except proline) and surrounding sequence context, assigning a potential outcome. NetOGlyc-4.0 predicts O-GalNAc glycosylation sites on serine and threonine residues using a neural network-based method and provides a prediction score.

PDK1(FASTA): >sp|O15530.1|PDPK1_HUMAN RecName: Full=3-phosphoinositide-dependent protein kinase 1; Short=hPDK1

MARTTSQLYDAVPIQSSVVLCSCPSPSMVRTQTESSTPPGIPGGSRQGPAMDGTAAEPRPGAGSLQHAQP

PPQPRKKRPEDFKFGKILGEGSFSTVVLARELATSREYAIKILEKRHIIKENKVPYVTRERDVMSRLDHP

FFVKLYFTFQDDEKLYFGLSYAKNGELLKYIRKIGSFDETCTRFYTAEIVSALEYLHGKGIIHRDLKPEN

ILLNEDMHIQITDFGTAKVLSPESKQARANSFVGTAQYVSPELLTEKSACKSSDLWALGCIIYQLVAGLP

PFRAGNEYLIFQKIIKLEYDFPEKFFPKARDLVEKLLVLDATKRLGCEEMEGYGPLKAHPFFESVTWENL

HQQTPPKLTAYLPAMSEDDEDCYGNYDNLLSQFGCMQVSSSSSSHSLSASDTGLPQRSGSNIEQYIHDLD

SNSFELDLQFSEDEKRLLLEKQAGGNPWHQFVENNLILKMGPVDKRKGLFARRRQLLLTEGPHLYYVDPV

NKVLKGEIPWSQELRPEAKNFKTFFVHTPNRTYYLMDPSGNAHKWCRKIQEVWRQRYQSHPDAAVQ

**5.24. Identification of PDK1 glycosylation sites by PNGase F–assisted LC–MS/MS**

**1. In-gel Digestion**

Cultured prostate cancer C4-2 cells in 10-cm dishes were harvested for total protein extraction when they reached 80% confluence. PDK1 protein was immunoprecipitated using a PDK1-specific antibody in co-immunoprecipitation (Co-IP) assays. The immunoprecipitated complexes were separated by SDS–PAGE, and the specific PDK1 band (approximately 68 kDa) was visualized by Coomassie Blue staining. The position of PDK1 was confirmed by parallel Western blotting using the same antibody. The corresponding gel region containing the PDK1 protein was excised and subjected to in-gel digestion for mass spectrometric analysis.

Gel pieces were cut into small fragments and destained with 50 mM ammonium bicarbonate (NH₄HCO₃) containing 50% acetonitrile (ACN, v/v) until clear. The gel pieces were dehydrated with 100% ACN for 5 min, and the solution was removed. Reduction was performed by adding 10 mM dithiothreitol (DTT) and incubating at 37 °C for 60 min. After dehydration with 100% ACN again, the gel pieces were treated with 55 mM iodoacetamide (IAA) and incubated at room temperature in the dark for 45 min. The gel pieces were washed with 50 mM NH₄HCO₃ and dehydrated again with 100% ACN. Finally, they were rehydrated in 50 mM NH₄HCO₃ containing 10 ng/μL trypsin (Promega) and incubated on ice for 1 h. Excess solution was removed, and in-gel digestion was performed overnight at 37 °C.

Prior to LC–MS/MS analysis, the excised gel pieces containing immunoprecipitated PDK1 were treated with PNGase F overnight at 37 °C in ¹⁸O-labeled ammonium bicarbonate buffer to cleave N-linked glycans. During this process, glycosylated Asn residues were converted to Asp, producing a mass shift of +2.99 Da, which was used to identify N-glycosylation sites.

The resulting peptides were extracted sequentially with 50% ACN/5% formic acid and 100% ACN. The combined extracts were dried completely in a vacuum concentrator and stored at −20 °C for subsequent LC-MS/MS analysis.

**2. LC-MS/MS Analysis**

The dried peptide samples were dissolved in solvent A (0.1% formic acid and 2% acetonitrile in water) and analyzed using an EASY-nLC 1200 UPLC system (Thermo Scientific). Solvent B consisted of 0.1% formic acid and 90% acetonitrile. Peptides were separated on a reversed-phase analytical column (15 cm length, 75 μm inner diameter) with the following linear gradient: 0–16 min, 6–25% B; 16–22 min, 25–35% B; 22–26 min, 35–80% B; 26–30 min, 80% B. The flow rate was maintained at 450 nL/min.

Eluted peptides were ionized via a Nanospray Flex ion source (NSI) and analyzed using a Thermo Scientific™ Q Exactive HF-X mass spectrometer. The electrospray voltage was set to 2.1 kV. Both precursor and fragment ions were detected in the Orbitrap analyzer. The full MS scan range was m/z 350–1800, with a resolution of 60,000 for MS¹ and 15,000 for MS². Data-dependent acquisition (DDA) was employed, selecting the top 20 most intense precursor ions for fragmentation via higher-energy collisional dissociation (HCD) with a normalized collision energy (NCE) of 28%. The automatic gain control (AGC) target was set to 5 × 10⁴, the intensity threshold to 5 × 10³ ions/s, the maximum injection time to 200 ms, and the dynamic exclusion duration to 15 s.

**3. Data Processing**

Raw LC-MS/MS data were processed using Proteome Discoverer 2.4 (Thermo Scientific). Spectra were searched against a database containing the target PDK1 protein sequence. The enzyme was specified as Trypsin/P, allowing two missed cleavages.The precursor ion mass tolerance was set to 10 ppm, and the fragment ion mass tolerance to 0.02 Da. Carbamidomethylation on cysteine was set as a fixed modification.Variable modifications included oxidation (Met), protein N-terminal acetylation, deamidation (¹⁸O)(Asn) to reflect PNGase F–induced deglycosylation and N-linked glycosylation sites, and O-glycosylation (Ser, Thr).Peptide identifications were filtered at high confidence with an ion score > 20.

Supporting Information


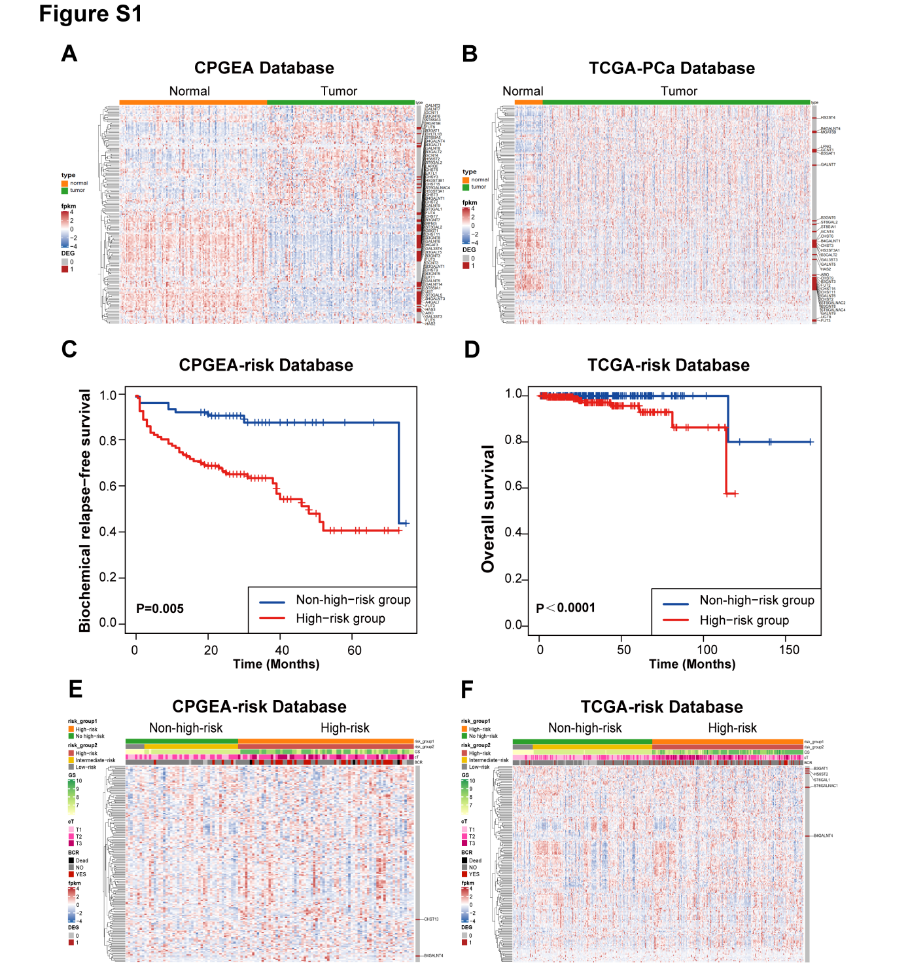


**Figure S1. Glycogene *B4GALNT4* is identified to be associated with high risk prostate cancer. A-B)** Heatmaps of differentially expressed glycogenes between tumor and normal tissues from the CPGEA database (A) (T=136, N=136) and TCGA-PCa database (B) (T=499, N=52). **C-D)** Kaplan-Meier curves for PCa patients in the non-high-risk group versus the high-risk group (C, b-RFS, *P*=0.005, CPGEA database; D, OS, *P*<0.0001, TCGA-risk database). **E-F)** Heatmaps of differentially expressed glycogenes between non-high-risk (NHR) and high-risk (HR) groups of PCa patients from CPGEA (NHR=48, HR=75) and TCGA-risk databases (NHR=194, HR=210).


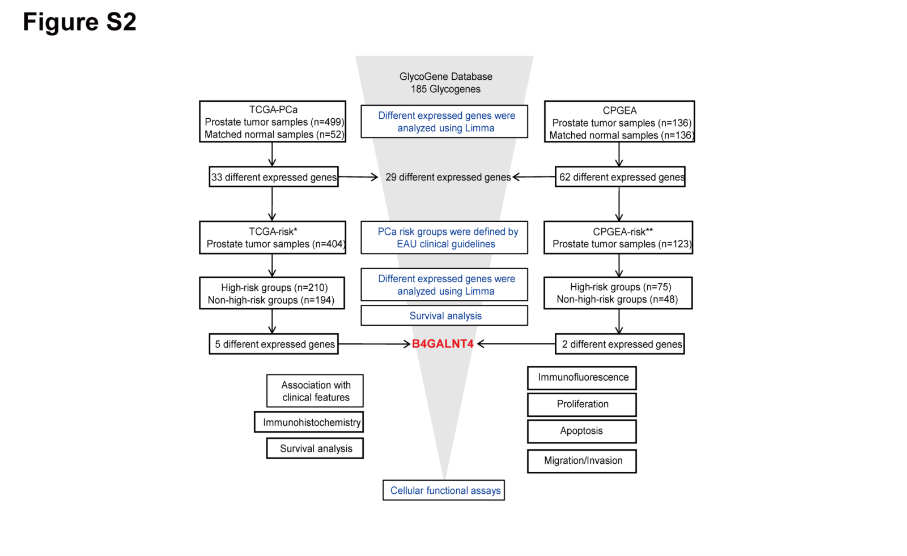


**Figure S2. Flowchart for the analysis in this study. 1)** Analysis of 185 glycogenes in TCGA-PCa and CPGEA databases identified 29 common differentially expressed glycogenes between tumor and normal; **2)** Integration of Limma analysis and survival analysis revealed *B4GALNT4* as the only significantly differentially expressed glycogene in prostate tumor tissues and in PCa patients at different risk groups; **3)** Clinical correlation analysis, immunohistochemistry, and survival analysis investigated the correlation between *B4GALNT4* expression and prognosis in PCa patients; **4)** A series of functional experiments were developed to assess the role of glycosyltransferase B4GALNT4 in the malignant phenotypes of PCa cells. * 95 samples were excluded due to missing clinical information in TCGA-risk database. ** 13 samples were excluded due to missing clinical information in CPGEA-risk database.


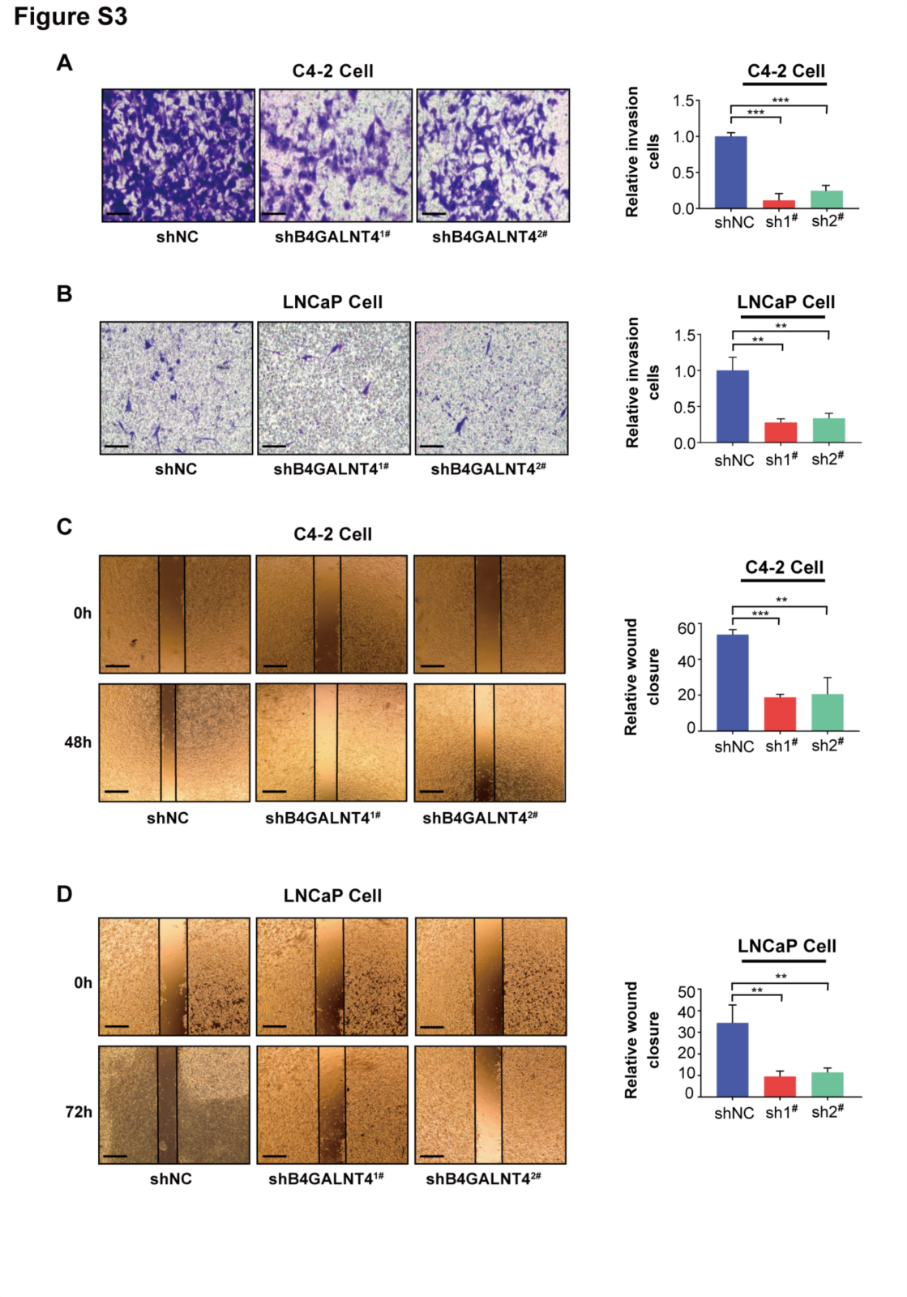


**Figure S3. Down-regulating *B4GALNT4* inhibits the invasive and migratory abilities of PCa cells. A-B)** Transwell assay assessed invasion levels of prostate tumor C4-2 cells (A) or LNCaP cells (B) in the shNC and *B4GALNT4* knockdown (shB4GALNT4^1#^, shB4GALNT4^2#^) groups. Representative invasion images and corresponding statistical analyses were shown (Scale bar: 50 μm). **C-D)** Wound healing assay evaluated migration ability of C4-2 cells (C) or LNCaP cells (D) in the control shNC and *B4GALNT4* knockdown groups (shB4GALNT4^1#^, shB4GALNT4^2#^). Representative migration images and corresponding statistical analyses were shown (Scale bar: 50 μm). Experiments were conducted in triplicate, and date are presented as mean ± SD (*, *P* < 0.05; * *, *P* < 0.01; * * *, *P* < 0.001; NS, *P* > 0.05).


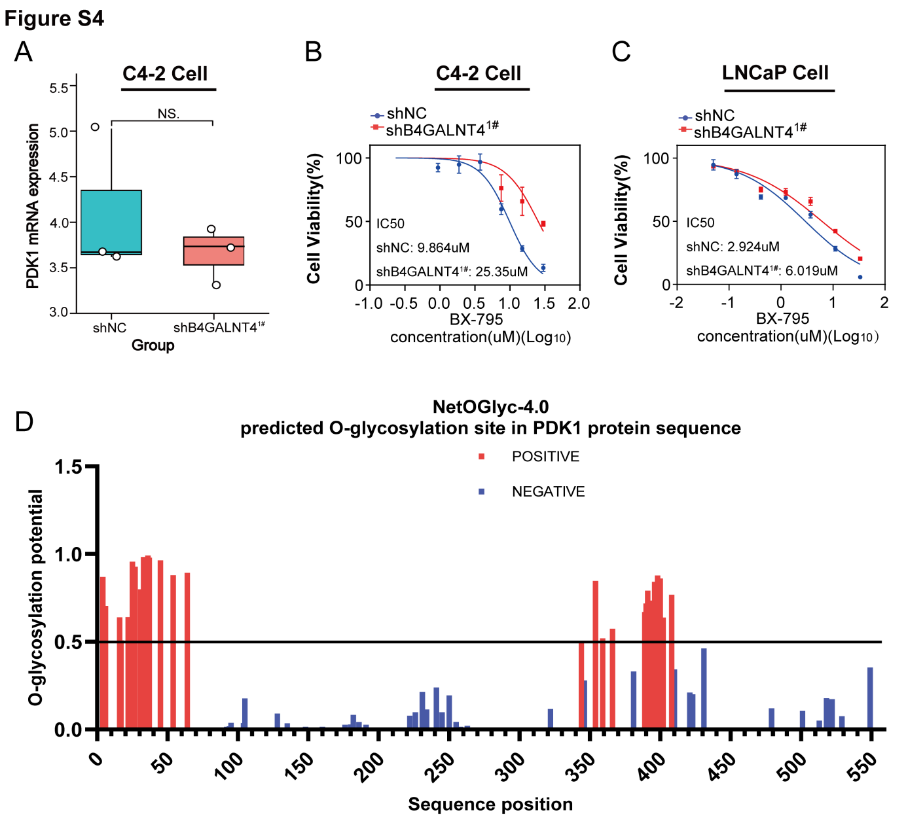


**Figure S4. Cell proliferation assay of PCa cells after knocking down *B4GALNT4*, and online prediction of O-glycosylation sites of PDK1.** **A)** RNA-seq data showing no significant difference in PDK1 mRNA expression between shNC and shB4GALNT4 cells; **B-C)** shNC and shB4GALNT4^1#^-transduced prostate cancer C4-2 cells (B) or LNCaP cells (C) were treated with various concentrations of the PDK1-specific inhibitor BX-795 for 72 hours. The relative cell viability of the tumor cells was detected at specific time points by CCK-8. **D)** NetOGlyc-4.0, an online protein database prediction tool, was designed to predict the O-glycosylation sites of PDK1 proteins. Red lines indicate high probability of O-glycosylation sites. Blue lines indicate negative sites.

**Table S1.** The 185 glycogenes were obtained from the glycogene database.

| **Entrez ID.** | **Gene symbol** | **Entry** | **Entrez ID.** | **Gene symbol** | **Entry** | **Entrez ID.** | **Gene symbol** | **Entry** | **Entrez ID.** | **Gene symbol** | **Entry** |
| --- | --- | --- | --- | --- | --- | --- | --- | --- | --- | --- | --- |
| 3038 | HAS3 | O00219 | 2527 | FUT5 | Q11128 | 256435 | ST6GALNAC3 | Q8NDV1 | 79147 | FKRP | Q9H9S5 |
| 4242 | MFNG | O00587 | 2529 | FUT7 | Q11130 | 3955 | LFNG | Q8NES3 | 50614 | GALNT9 | Q9HCQ5 |
| 9957 | HS3ST1 | O14792 | 6482 | ST3GAL1 | Q11201 | 166012 | CHST13 | Q8NET6 | 79868 | ALG13 | Q9NP73 |
| 8473 | OGT | O15294 | 6487 | ST3GAL3 | Q11203 | 93010 | B3GNT7 | Q8NFL0 | 53947 | A4GALT | Q9NPC4 |
| 29906 | ST8SIA5 | O15466 | 6484 | ST3GAL4 | Q11206 | 124872 | B4GALNT2 | Q8NHY0 | 50515 | CHST11 | Q9NPF2 |
| 51046 | ST8SIA3 | O43173 | 2591 | GALNT3 | Q14435 | 347734 | SLC35B2 | Q8TB61 | 135152 | B3GAT2 | Q9NPZ5 |
| 9334 | B4GALT5 | O43286 | 2131 | EXT1 | Q16394 | 55790 | CSGALNACT1 | Q8TDX6 | 55501 | CHST12 | Q9NRB3 |
| 11041 | B3GNT1 | O43505 | 7357 | UGCG | Q16739 | 55624 | POMGNT1 | Q8WZA1 | 56913 | C1GALT1 | Q9NS00 |
| 9486 | CHST10 | O43529 | 6483 | ST3GAL2 | Q16842 | 6489 | ST8SIA1 | Q92185 | 56548 | CHST7 | Q9NS84 |
| 8707 | B3GALT2 | O43825 | 7368 | UGT8 | Q16880 | 8128 | ST8SIA2 | Q92186 | 55808 | ST6GALNAC1 | Q9NSC7 |
| 2137 | EXTL3 | O43909 | 440138 | ALG11 | Q2TAA5 | 7903 | ST8SIA4 | Q92187 | 23169 | SLC35D1 | Q9NTN3 |
| 8534 | CHST1 | O43916 | 146664 | MGAT5B | Q3V5L5 | 10195 | ALG3 | Q92685 | 26290 | GALNT8 | Q9NY28 |
| 9394 | HS6ST1 | O60243 | 170384 | FUT11 | Q495W5 | 3037 | HAS2 | Q92819 | 55757 | UGGT2 | Q9NYU1 |
| 8703 | B4GALT3 | O60512 | 84920 | ALG10 | Q5BKT4 | 3036 | HAS1 | Q92839 | 56886 | UGGT1 | Q9NYU2 |
| 8702 | B4GALT4 | O60513 | 64409 | WBSCR17 | Q6IS24 | 2134 | EXTL1 | Q92935 | 51301 | GCNT4 | Q9P109 |
| 8813 | DPM1 | O60762 | 283358 | B4GALNT3 | Q6L9W6 | 2132 | EXT2 | Q93063 | 54480 | CHPF2 | Q9P2E5 |
| 8704 | B4GALT2 | O60909 | 84750 | FUT10 | Q6P4F1 | 84912 | SLC35B4 | Q969S0 | 27087 | B3GAT1 | Q9P2W7 |
| 2218 | FKTN | O75072 | 145173 | B3GALTL | Q6Y288 | 30815 | ST6GALNAC6 | Q969X2 | 54344 | DPM3 | Q9P2X0 |
| 8706 | B3GALNT1 | O75752 | 192134 | B3GNT6 | Q6ZMB0 | 89792 | GAL3ST3 | Q96A11 | 2135 | EXTL2 | Q9UBQ6 |
| 26229 | B3GAT3 | O94766 | 337876 | CHSY3 | Q70JA7 | 55343 | SLC35C1 | Q96A29 | 11285 | B4GALT7 | Q9UBV7 |
| 8818 | DPM2 | O94777 | 11046 | SLC35D2 | Q76EJ3 | 29071 | C1GALT1C1 | Q96EU7 | 9331 | B4GALT6 | Q9UBX8 |
| 9245 | GCNT3 | O95395 | 338707 | B4GALNT4 | Q76KP1 | 199857 | ALG14 | Q96F25 | 10610 | ST6GALNAC2 | Q9UJ37 |
| 9215 | LARGE | O95461 | 83539 | CHST9 | Q7L1S5 | 79623 | GALNT14 | Q96FL9 | 29954 | POMT2 | Q9UKY4 |
| 9348 | NDST3 | O95803 | 51363 | CHST15 | Q7LFX5 | 84620 | ST6GAL2 | Q96JF0 | 11320 | MGAT4A | Q9UM21 |
| 8705 | B3GALT4 | O96024 | 9653 | HS2ST1 | Q7LGA3 | 126792 | B3GALT6 | Q96L58 | 51146 | A4GNT | Q9UNA3 |
| 2683 | B4GALT1 | P15291 | 9469 | CHST3 | Q7LGC8 | 90161 | HS6ST2 | Q96MM7 | 8869 | ST3GAL5 | Q9UNP4 |
| 6480 | ST6GAL1 | P15907 | 374907 | B3GNT8 | Q7Z7M8 | 79690 | GAL3ST4 | Q96RP7 | 11282 | MGAT4B | Q9UQ53 |
| 28 | ABO | P16442 | 11227 | GALNT5 | Q7Z7M9 | 9514 | GAL3ST1 | Q99999 | 10690 | FUT9 | Q9Y231 |
| 2523 | FUT1 | P19526 | 51809 | GALNT7 | Q86SF2 | 56052 | ALG1 | Q9BT22 | 10402 | ST3GAL6 | Q9Y274 |
| 2525 | FUT3 | P21217 | 55568 | GALNT10 | Q86SR1 | 79087 | ALG12 | Q9BV10 | 9956 | HS3ST2 | Q9Y278 |
| 2526 | FUT4 | P22083 | 22856 | CHSY1 | Q86X52 | 81849 | ST6GALNAC5 | Q9BVH7 | 10331 | B3GNT3 | Q9Y2A9 |
| 4245 | MGAT1 | P26572 | 64131 | XYLT1 | Q86Y38 | 79053 | ALG8 | Q9BVK2 | 10090 | UST | Q9Y2C2 |
| 2528 | FUT6 | P51993 | 79695 | GALNT12 | Q8IXK2 | 2530 | FUT8 | Q9BYC5 | 10317 | B3GALT5 | Q9Y2C3 |
| 3340 | NDST1 | P52848 | 79586 | CHPF | Q8IZ52 | 84002 | B3GNT5 | Q9BYG0 | 23443 | SLC35A3 | Q9Y2D2 |
| 8509 | NDST2 | P52849 | 266722 | HS6ST3 | Q8IZP7 | 79369 | B3GNT4 | Q9C0J1 | 23275 | POFUT2 | Q9Y2G5 |
| 338596 | ST8SIA6 | P61647 | 222537 | HS3ST5 | Q8IZT8 | 23563 | CHST5 | Q9GZS9 | 9435 | CHST2 | Q9Y4C5 |
| 7355 | SLC35A2 | P78381 | 2651 | GCNT2 | Q8N0V5 | 4166 | CHST6 | Q9GZX3 | 8708 | B3GALT1 | Q9Y5Z6 |
| 10559 | SLC35A1 | P78382 | 117248 | GALNT15 | Q8N3T1 | 64132 | XYLT2 | Q9H1B5 | 5986 | RFNG | Q9Y644 |
| 10237 | SLC35B1 | P78383 | 120071 | GYLTL1B | Q8N3Y3 | 51000 | SLC35B3 | Q9H1N7 | 9951 | HS3ST4 | Q9Y661 |
| 2583 | B4GALNT1 | Q00973 | 8693 | GALNT4 | Q8N4A0 | 64377 | CHST8 | Q9H2A9 | 9953 | HS3ST3B1 | Q9Y662 |
| 2650 | GCNT1 | Q02742 | 26301 | GBGT1 | Q8N5D6 | 1798 | DPAGT1 | Q9H3H5 | 9955 | HS3ST3A1 | Q9Y663 |
| 4248 | MGAT3 | Q09327 | 55454 | CSGALNACT2 | Q8N6G5 | 64090 | GAL3ST2 | Q9H3Q3 | 29929 | ALG6 | Q9Y672 |
| 4249 | MGAT5 | Q09328 | 10164 | CHST4 | Q8NCG5 | 64579 | NDST4 | Q9H3R1 | 29880 | ALG5 | Q9Y673 |
| 4247 | MGAT2 | Q10469 | 113189 | CHST14 | Q8NCH0 | 23509 | POFUT1 | Q9H488 | 10585 | POMT1 | Q9Y6A1 |
| 2590 | GALNT2 | Q10471 | 11226 | GALNT6 | Q8NCL4 | 27090 | ST6GALNAC4 | Q9H4F1 |  |  |  |
| 2589 | GALNT1 | Q10472 | 148789 | B3GALNT2 | Q8NCR0 | 85365 | ALG2 | Q9H553 |  |  |  |
| 2524 | FUT2 | Q10981 | 63917 | GALNT11 | Q8NCW6 | 79796 | ALG9 | Q9H6U8 |  |  |  |

**Table S2.** The 29 differentially expressed glycogenes shared between TCGA-PCa and CPGEA.

| **Gene Symbol** | **TCGA-PCa Database** | | | **CPGEA Database** | | |
| --- | --- | --- | --- | --- | --- | --- |
|  | **logFC** | ***P.*Value** | **adj*.P*.Val** | **logFC** | ***P*.Value** | **adj.*P.*Val** |
| B4GALNT4 | 1.717 | 3.76E-20 | 8.50E-19 | 0.702 | 1.51E-08 | 2.90E-08 |
| GALNT7 | 1.265 | 6.38E-18 | 1.05E-16 | 1.420 | 2.70E-42 | 4.95E-41 |
| GCNT1 | 1.700 | 2.54E-17 | 3.83E-16 | 1.426 | 3.38E-24 | 1.55E-23 |
| B3GAT1 | 1.906 | 3.51E-17 | 4.88E-16 | 1.112 | 1.61E-10 | 3.63E-10 |
| MGAT5B | 1.512 | 2.84E-11 | 1.43E-10 | 0.718 | 9.00E-06 | 1.46E-05 |
| B3GNT6 | 2.233 | 4.56E-06 | 1.13E-05 | 1.094 | 0.000994375 | 0.001389089 |
| GCNT4 | -2.251 | 1.31E-34 | 2.37E-32 | -1.648 | 4.71E-45 | 9.58E-44 |
| ST6GALNAC4 | -1.451 | 1.06E-26 | 6.38E-25 | -1.563 | 3.22E-50 | 1.18E-48 |
| B3GNT8 | -1.675 | 2.86E-21 | 8.64E-20 | -1.252 | 6.29E-36 | 7.67E-35 |
| CHST2 | -1.500 | 1.23E-18 | 2.47E-17 | -1.956 | 3.43E-51 | 1.57E-49 |
| CHST3 | -1.198 | 4.30E-16 | 5.56E-15 | -1.657 | 4.69E-50 | 1.43E-48 |
| FUT3 | -2.266 | 5.72E-16 | 6.90E-15 | -0.808 | 0.000202768 | 0.00030168 |
| B3GALT2 | -1.806 | 1.61E-15 | 1.72E-14 | -0.831 | 9.23E-36 | 1.06E-34 |
| GALNT9 | -1.771 | 8.61E-15 | 8.20E-14 | -1.536 | 6.94E-18 | 2.27E-17 |
| CHST11 | -1.092 | 9.38E-15 | 8.49E-14 | -1.134 | 7.52E-24 | 3.28E-23 |
| GALNT6 | -1.112 | 1.12E-13 | 8.82E-13 | -1.296 | 1.26E-25 | 6.76E-25 |
| CHST15 | -1.101 | 2.21E-13 | 1.60E-12 | -1.385 | 8.61E-42 | 1.43E-40 |
| ABO | -1.971 | 3.03E-13 | 2.03E-12 | -3.098 | 1.89E-46 | 4.94E-45 |
| CHST6 | -1.394 | 3.31E-13 | 2.14E-12 | -1.656 | 5.85E-30 | 4.12E-29 |
| HS3ST3A1 | -1.410 | 6.57E-12 | 3.60E-11 | -1.901 | 2.82E-54 | 2.58E-52 |
| FUT2 | -1.414 | 7.03E-12 | 3.74E-11 | -1.738 | 3.29E-35 | 3.55E-34 |
| CHST9 | -1.964 | 3.57E-11 | 1.75E-10 | -1.962 | 1.25E-28 | 7.62E-28 |
| ST8SIA1 | -1.112 | 1.83E-10 | 8.72E-10 | -1.481 | 2.81E-33 | 2.45E-32 |
| B4GALNT1 | -1.015 | 2.07E-10 | 9.61E-10 | -1.889 | 1.38E-51 | 8.40E-50 |
| B3GNT3 | -1.735 | 2.44E-08 | 7.61E-08 | -1.721 | 1.83E-16 | 5.41E-16 |
| ST6GAL2 | -1.160 | 1.43E-07 | 4.24E-07 | -1.706 | 1.83E-32 | 1.53E-31 |
| GALNT5 | -1.223 | 2.49E-07 | 6.94E-07 | -2.332 | 2.06E-54 | 2.58E-52 |
| GAL3ST3 | -1.020 | 3.12E-06 | 8.06E-06 | -0.830 | 5.13E-05 | 7.95E-05 |
| HAS2 | -1.002 | 4.41E-06 | 1.11E-05 | -0.852 | 6.48E-10 | 1.38E-09 |

logFC, log fold change; adj.P.Val, adjusted p-value.

**Table S3.** 2023 EAU PCa risk classification criteria

| **Low-risk** | **Intermediate-risk** | **High-risk** | |
| --- | --- | --- | --- |
| PSA < 10 ng/mL | PSA 10–20 ng/mL | PSA > 20 ng/mL | any PSA |
| and GS < 7  (ISUP grade 1) | or GS 7  (ISUP grade 2/3) | or GS > 7  (ISUP grade 4/5) | any GS  (any ISUP grade) |
| and cT1-2a  Localised | or cT2b  Localised | or cT2c  Localised | cT3-4 or cN+  Locally advanced |

EAU, European Association of Urology; GS, Gleason score; ISUP, International Society for Urological Pathology; PSA, prostate-specific antigen.

**Table S4.** Differentially expressed glycogenes between high-risk and non-high-risk prostate cancer samples in the TCGA-risk and CPGEA-risk databases**.**

| **Gene Symbol** | **TCGA-risk Database** | | | **Gene Symbol** | **CPGEA-risk Database** | | |
| --- | --- | --- | --- | --- | --- | --- | --- |
|  | **logFC** | ***P.*Value** | **adj.*P*.Val** |  | **logFC** | ***P*.Value** | **adj.*P*.Val** |
| **B4GALNT4** | 0.502 | 5.67E-05 | 0.000790121 | **B4GALNT4** | 0.736 | 0.00068986 | 0.096915135 |
| B3GAT1 | -0.776 | 4.40E-08 | 1.99E-06 | CHST13 | 0.848 | 0.001059182 | 0.096915135 |
| ST6GALNAC1 | -0.678 | 9.89E-07 | 2.24E-05 |  |  |  |  |
| ST6GAL1 | -0.581 | 3.04E-05 | 0.000458471 |  |  |  |  |
| HS6ST2 | -0.560 | 0.00443329 | 0.020582457 |  |  |  |  |

logFC, log fold change; ;adj.P.Val, adjusted p-value.

**Table S5.** Univariate and multivariate analyses of the correlation between *B4GALNT4* expression and b-RFS in prostate cancer patients using TCGA-PCa database.

| **Parameter** | **Univariate analysis** | | | **Multivariate analysis** | | |
| --- | --- | --- | --- | --- | --- | --- |
|  | **HR** | **95%CI** | ***P*** | **HR** | **95%CI** | ***P*** |
| Age | 1.015 | 0.981-1.050 | 0.378 | / | / | / |
| Gleason score | 1.978 | 1.546-2.529 | **0.000** | 1.587 | 1.183 -2.131 | **0.002** |
| Surgical margin | 2.177 | 1.398-3.389 | **0.001** | 1.180 | 0.713 -1.955 | 0.520 |
| Lymph nodes | 1.808 | 1.087-3.007 | **0.023** | 0.870 | 0.502 -1.509 | 0.620 |
| Pathological T stage | 2.644 | 1.657-4.220 | **0.000** | 1.637 | 0.923 -2.903 | 0.092 |
| B4GALNT4 | 1.586 | 1.277-1.971 | **0.000** | 1.324 | 1.046 -1.676 | **0.019** |

Bold values indicate *P* <0.05. b-RFS, biochemical relapse-free survival; HR, hazard ratio; CI, confidence interval. Not available (NA) of surgical margin:15; NA of Lymph nodes: 73; NA of pathological T stage: 7.

**Table S6.** Univariate and multivariate analyses of the correlation between *B4GALNT4* expression and b-RFS in prostate cancer patients using CPGEA data.

| **Parameter** | **Univariate analysis** | | | **Multivariate analysis** | | |
| --- | --- | --- | --- | --- | --- | --- |
|  | **HR** | **95%CI** | ***P*** | **HR** | **95%CI** | ***P*** |
| Age | 1.011 | 0.960-1.064 | 0.690 | / | / | / |
| PSA | 1.003 | 1.001-1.004 | **0.000** | 1.003 | 1.001 -1.004 | **0.001** |
| Bone Metastasis | 2.933 | 1.028-8.370 | **0.044** | 3.833 | 1.280 -11.476 | **0.016** |
| Gleason score | 1.661 | 1.182-2.335 | **0.003** | 1.300 | 0.884 -1.912 | 0.183 |
| Pathological T Stage | 1.946 | 1.088-3.480 | **0.025** | 1.655 | 0.905 -3.029 | 0.102 |
| Extraprostatic  invasion | 1.429 | 0.724-2.820 | 0.304 | / | / | / |
| Surgical margin | 1.899 | 0.967-3.730 | 0.063 | 1.824 | 0.895 -3.720 | 0.098 |
| B4GALNT4 | 1.823 | 1.222-2.721 | **0.003** | 1.652 | 1.074 -2.541 | **0.022** |

Bold values indicate P <0.05. b-RFS, biochemical relapse-free survival; HR, hazard ratio; CI, confidence interval; PSA, prostate-specific antigen. Not available (NA) of Gleason score: 1; NA of Extraprostatic invasion: 2; NA of Surgical margin: 1.

**Table S7.** IHC staining analysis of B4GALNT4 protein expression in prostate cancer tissues.

| **Subgroup** | | **B4GALNT4 protein expression** | | **χ^2^ Value** | ***p*-Value** |
| --- | --- | --- | --- | --- | --- |
|  |  | **High** | **Low** |  |  |
| Overall | | 25/67 | 42/67 |  |  |
| Age* | ≤69 year | 12/35 | 23/35 |  |  |
|  | ＞69 year | 13/32 | 19/32 | 0.287 | 0.592 |
| PSA** | ≤20 ng/ml | 8/33 | 25/33 |  |  |
|  | ＞20 ng/ml | 17/34 | 17/34 | 4.750 | **0.029** |
| Gleason score | ≤7 | 9/42 | 33/42 |  |  |
|  | ＞7 | 16/25 | 9/25 | 12.143 | **0.000** |
| Surgical margin | positive | 11/22 | 11/22 |  |  |
|  | negative | 14/45 | 31/45 | 2.254 | 0.133 |
| Extraprostatic invasion | positive | 16/32 | 16/32 |  |  |
|  | negative | 9/35 | 26/35 | 4.215 | **0.040** |
| Seminal Vesical | positive | 8/12 | 4/12 |  |  |
|  | negative | 17/55 | 38/55 | 5.385 | **0.020** |
| Pathological T stage | pT2 | 6/31 | 25/31 |  |  |
|  | pT3, pT4 | 19/36 | 17/36 | 7.955 | **0.005** |

Bold values indicate P <0.05. *, Data were grouped according to the median value of age; **, Data were grouped according to the median value of PSA level; IHC, immunohistochemistry; PSA, prostate-specific antigen.

**Table S8.** RNA-sequencing results (shNC vs. sh1^#^)

**Table S9.** Proteomic profiling results (shNC vs. sh1^#^)

**Table S10.** PDK1 glycosylation site prediction

| **Glycosylation type** | **Seqname** | **Start** | **End** | **Score** | **Comment** |
| --- | --- | --- | --- | --- | --- |
| **NetNGlyc-1.0**  **N-linked glycosylation** | NP_002604_1 | 122 | 122 | 0.199 | NEGATIVE |
|  | NP_002604_1 | 164 | 164 | 0.5307 | POSITIVE |
|  | NP_002604_1 | 210 | 210 | 0.7457 | POSITIVE |
|  | NP_002604_1 | 214 | 214 | 0.725 | POSITIVE |
|  | NP_002604_1 | 240 | 240 | 0.5492 | POSITIVE |
|  | NP_002604_1 | 286 | 286 | 0.763 | POSITIVE |
|  | NP_002604_1 | 349 | 349 | 0.6067 | POSITIVE |
|  | NP_002604_1 | 375 | 375 | 0.566 | POSITIVE |
|  | NP_002604_1 | 378 | 378 | 0.7006 | POSITIVE |
|  | NP_002604_1 | 411 | 411 | 0.646 | POSITIVE |
|  | NP_002604_1 | 422 | 422 | 0.4849 | NEGATIVE |
|  | NP_002604_1 | 446 | 446 | 0.575 | POSITIVE |
|  | NP_002604_1 | 454 | 454 | 0.3552 | NEGATIVE |
|  | NP_002604_1 | 455 | 455 | 0.5298 | POSITIVE |
|  | NP_002604_1 | 491 | 491 | 0.4513 | NEGATIVE |
|  | NP_002604_1 | 510 | 510 | 0.5517 | POSITIVE |
|  | NP_002604_1 | 520 | 520 | 0.5131 | POSITIVE |
|  | NP_002604_1 | 531 | 531 | 0.5187 | POSITIVE |
| **NetOGlyc-4.0**  **O-linked glycosylation** | NP_002604_1 | 4 | 4 | 0.869954 | POSITIVE |
|  | NP_002604_1 | 5 | 5 | 0.654292 | POSITIVE |
|  | NP_002604_1 | 6 | 6 | 0.70472 | POSITIVE |
|  | NP_002604_1 | 16 | 16 | 0.639711 | POSITIVE |
|  | NP_002604_1 | 17 | 17 | 0.508045 | POSITIVE |
|  | NP_002604_1 | 22 | 22 | 0.641043 | POSITIVE |
|  | NP_002604_1 | 25 | 25 | 0.957196 | POSITIVE |
|  | NP_002604_1 | 27 | 27 | 0.927859 | POSITIVE |
|  | NP_002604_1 | 31 | 31 | 0.799472 | POSITIVE |
|  | NP_002604_1 | 33 | 33 | 0.983238 | POSITIVE |
|  | NP_002604_1 | 35 | 35 | 0.980771 | POSITIVE |
|  | NP_002604_1 | 36 | 36 | 0.991872 | POSITIVE |
|  | NP_002604_1 | 37 | 37 | 0.979314 | POSITIVE |
|  | NP_002604_1 | 45 | 45 | 0.963949 | POSITIVE |
|  | NP_002604_1 | 54 | 54 | 0.879989 | POSITIVE |
|  | NP_002604_1 | 64 | 64 | 0.892881 | POSITIVE |
|  | NP_002604_1 | 92 | 92 | 0.013249 | NEGATIVE |
|  | NP_002604_1 | 94 | 94 | 0.0185663 | NEGATIVE |
|  | NP_002604_1 | 95 | 95 | 0.0377157 | NEGATIVE |
|  | NP_002604_1 | 104 | 104 | 0.0371553 | NEGATIVE |
|  | NP_002604_1 | 105 | 105 | 0.177113 | NEGATIVE |
|  | NP_002604_1 | 128 | 128 | 0.090169 | NEGATIVE |
|  | NP_002604_1 | 135 | 135 | 0.0355765 | NEGATIVE |
|  | NP_002604_1 | 148 | 148 | 0.00454779 | NEGATIVE |
|  | NP_002604_1 | 160 | 160 | 0.00630516 | NEGATIVE |
|  | NP_002604_1 | 176 | 176 | 0.0274581 | NEGATIVE |
|  | NP_002604_1 | 180 | 180 | 0.0285213 | NEGATIVE |
|  | NP_002604_1 | 182 | 182 | 0.0829018 | NEGATIVE |
|  | NP_002604_1 | 186 | 186 | 0.0415482 | NEGATIVE |
|  | NP_002604_1 | 191 | 191 | 0.026765 | NEGATIVE |
|  | NP_002604_1 | 222 | 222 | 0.0777161 | NEGATIVE |
|  | NP_002604_1 | 226 | 226 | 0.0977 | NEGATIVE |
|  | NP_002604_1 | 231 | 231 | 0.213335 | NEGATIVE |
|  | NP_002604_1 | 234 | 234 | 0.114287 | NEGATIVE |
|  | NP_002604_1 | 241 | 241 | 0.239632 | NEGATIVE |
|  | NP_002604_1 | 245 | 245 | 0.0979352 | NEGATIVE |
|  | NP_002604_1 | 250 | 250 | 0.19421 | NEGATIVE |
|  | NP_002604_1 | 255 | 255 | 0.0425414 | NEGATIVE |
|  | NP_002604_1 | 258 | 258 | 0.00730011 | NEGATIVE |
|  | NP_002604_1 | 262 | 262 | 0.0151703 | NEGATIVE |
|  | NP_002604_1 | 263 | 263 | 0.0211215 | NEGATIVE |
|  | NP_002604_1 | 322 | 322 | 0.116557 | NEGATIVE |
|  | NP_002604_1 | 344 | 344 | 0.505947 | POSITIVE |
|  | NP_002604_1 | 346 | 346 | 0.27894 | NEGATIVE |
|  | NP_002604_1 | 354 | 354 | 0.84709 | POSITIVE |
|  | NP_002604_1 | 359 | 359 | 0.519114 | POSITIVE |
|  | NP_002604_1 | 366 | 366 | 0.573826 | POSITIVE |
|  | NP_002604_1 | 381 | 381 | 0.331198 | NEGATIVE |
|  | NP_002604_1 | 389 | 389 | 0.669831 | POSITIVE |
|  | NP_002604_1 | 390 | 390 | 0.718133 | POSITIVE |
|  | NP_002604_1 | 391 | 391 | 0.791866 | POSITIVE |
|  | NP_002604_1 | 392 | 392 | 0.552211 | POSITIVE |
|  | NP_002604_1 | 393 | 393 | 0.62898 | POSITIVE |
|  | NP_002604_1 | 394 | 394 | 0.734272 | POSITIVE |
|  | NP_002604_1 | 396 | 396 | 0.842421 | POSITIVE |
|  | NP_002604_1 | 398 | 398 | 0.878692 | POSITIVE |
|  | NP_002604_1 | 400 | 400 | 0.860619 | POSITIVE |
|  | NP_002604_1 | 402 | 402 | 0.638061 | POSITIVE |
|  | NP_002604_1 | 408 | 408 | 0.767856 | POSITIVE |
|  | NP_002604_1 | 410 | 410 | 0.342365 | NEGATIVE |
|  | NP_002604_1 | 421 | 421 | 0.210417 | NEGATIVE |
|  | NP_002604_1 | 423 | 423 | 0.201183 | NEGATIVE |
|  | NP_002604_1 | 431 | 431 | 0.461735 | NEGATIVE |
|  | NP_002604_1 | 479 | 479 | 0.120497 | NEGATIVE |
|  | NP_002604_1 | 501 | 501 | 0.105899 | NEGATIVE |
|  | NP_002604_1 | 513 | 513 | 0.0512012 | NEGATIVE |
|  | NP_002604_1 | 518 | 518 | 0.177981 | NEGATIVE |
|  | NP_002604_1 | 522 | 522 | 0.173055 | NEGATIVE |
|  | NP_002604_1 | 529 | 529 | 0.0754097 | NEGATIVE |
|  | NP_002604_1 | 549 | 549 | 0.352862 | NEGATIVE |

**Table S11.** Full list of PDK1-derived peptides identified by immunoprecipitation-mass spectrometry

**Table S12.** Mass spectrometric evidence for N-glycosylation at N531 of PDK1.

| **Accession** | FE559GP |
| --- | --- |
| **Protein names** | PDK1 |
| **MW [kDa]** | 63.10 |
| **Protein score** | 1495 |
| **Sequence coverage (%)** | 59.00 |
| **Position** | N531 |
| **Peptide score** | 43.00 |
| **Modified sequence** | TYYLMDPSGN(Ng)AHK |
| **Mass error [ppm]** | -0.69 |
| **# PSMs** | 2 |
| **Abundances** | 1169786.19 |

MW [kDa], PSMs, Number of Peptide-Spectrum Matches.

**Table S13.** List of reference databases for bioinformatics analysis of glycogenes.

| **Databases** | TCGA | CPGEA | SMMU | DKFZ |
| --- | --- | --- | --- | --- |
| **Samples** | Tumor:499 Normal:52 | Tumor:136 Normal:136 | Tumor:65 | Tumor:118 |
| **Type Detailed** | RNA | RNA | RNA | RNA |
| **AGE** | √ | √ | √ | √ |
| **Gleason Score** | √ | √ | √ | √ |
| **Gleason Group** | √ | √ | √ | √ |
| **Initial Diagnosis PSA** | × | √ | √ | √ |
| **cT** | √ | √ | × | × |
| **cN** | × | √ | × | × |
| **cM** | × | √ | × | × |
| **Bone Metastasis** | × | √ | × | × |
| **Metastasis** | × | × | × | √ |
| **（BCR)Biochemical recurrence indicator** | √ | √ | × | √ |
| **BCR Time** | √ | √ | × | √ |
| **pT** | √ | √ | √ | √ |
| **pN** | √ | √ | √ | × |
| **pM** | × | √ | √ | × |
| **Extraprostatic** | × | √ | √ | × |
| **Seminal Vesical** | × | √ | √ | × |
| **Lymph Node Metastasis** | × | × | √ | × |
| **Bladder Neck Invasion** | × | × | √ | × |
| **Surgical Margin** | × | √ | × | × |
| **OS Status** | √ | × | × | × |
| **OS Time** | √ | × | × | × |
| **DFS Status** | √ | × | × | × |
| **DFS Time** | √ | × | × | × |

TCGA, The Cancer Genome Atlas; CPGEA, Chinese Prostate Cancer Genome and Epigenome Atlas; SMMU, Second Military Medical University; DKFZ, German Cancer Research Center; RNA, Ribonucleic Acid; AGE, age; PSA, Prostate-Specific Antigen; cT, clinical T stage; cN, clinical N stage; cM, clinical M stage; BCR, Biochemical Recurrence; pT, pathological T stage; pN, pathological N stage; pM, pathological M stage; OS, Overall Survival; DFS, disease-free survival.

**Table S14.** Sequences of primers and oligonucleotides used for qPCR, gene knockdown (shRNA), and site-directed mutagenesis.

| **Name** | **Quantitative Real-Time PCR Primer** |
| --- | --- |
| B4GALNT4-F | 5’- ACTGGGAGCTCCTGGACA -3’ |
| B4GALNT4-R | 5’-TGGTGATAGAAATTCCGCAGT-3’ |
| PDK1-F | 5’- GGCCAGGACCACCAGCCA -3’ |
| PDK1-R | 5’- AGCCAGGACAACCGTGGAAA -3’ |
| Actin-F | 5’- CGCGAGAAGATGCCCAGATC -3 |
| Actin-R | 5’- TCACCGGAGTCCATCACGA -3’ |
| **Name** | **B4GALNT4 shRNA** |
| shB4GALNT4^1#^ | 5’- GCAATTTGTGTACCTGTCCTT -3’ |
| shB4GALNT4^2#^ | 5’- GGAGGTACTACTTTGAGTTGC -3’ |
| shNC | 5’- GUACCUUGACAGUACCGAUTT -3’ |
| **Name** | **Site Mutation PCR Primer** |
| HA-PDK1^N531A^-F | 5’- ATGGACCCCAGCGG G**GC**CGCACACAAGTGGTG -3’ |
| HA-PDK1^N531A^-R | 5’- CACCACTTGTGTGCG**GC**CCCGCTGGGGTCCAT -3’ |

F, forward; R, reverse.
